# COVID-19 in Africa: Underreporting, demographic effect, chaotic dynamics, and mitigation strategy impact

**DOI:** 10.1371/journal.pntd.0010735

**Published:** 2022-09-16

**Authors:** Nathan Thenon, Marisa Peyre, Mireille Huc, Abdoulaye Touré, François Roger, Sylvain Mangiarotti

**Affiliations:** 1 Centre d’Etudes Spatiales de la Biosphère, CESBIO/OMP, UMR UPS-CNES-CNRS-IRD-INRAe, Toulouse, France; 2 Animal Santé Territoires Risques Ecosystèmes, ASTRE/CIRAD, UMR CIRAD-INRAe-University of Montpellier, Montpellier, France; 3 Centre de Recherche et de Formation en Infectiologie de Guinée, Université Gamal Abdel Nasser de Conakry, Conakry, Guinea; 4 Institut National de Santé Publique, Conakry, Guinea; KU Leuven, BELGIUM

## Abstract

The epidemic of COVID-19 has shown different developments in Africa compared to the other continents. Three different approaches were used in this study to analyze this situation. In the first part, basic statistics were performed to estimate the contribution of the elderly people to the total numbers of cases and deaths in comparison to the other continents; Similarly, the health systems capacities were analysed to assess the level of underreporting. In the second part, differential equations were reconstructed from the epidemiological time series of cases and deaths (from the *John Hopkins University*) to analyse the dynamics of COVID-19 in seventeen countries. In the third part, the time evolution of the contact number was reconstructed since the beginning of the outbreak to investigate the effectiveness of the mitigation strategies. Results were compared to the *Oxford stringency index* and to the mobility indices of the *Google Community Mobility Reports*.

Compared to Europe, the analyses show that the lower proportion of elderly people in Africa enables to explain the lower total numbers of cases and deaths by a factor of 5.1 on average (from 1.9 to 7.8). It corresponds to a genuine effect. Nevertheless, COVID-19 numbers are effectively largely underestimated in Africa by a factor of 8.5 on average (from 1.7 to 20. and more) due to the weakness of the health systems at country level. Geographically, the models obtained for the dynamics of cases and deaths reveal very diversified dynamics. The dynamics is chaotic in many contexts, including a situation of bistability rarely observed in dynamical systems. Finally, the contact number directly deduced from the epidemiological observations reveals an effective role of the mitigation strategies on the short term. On the long term, control measures have contributed to maintain the epidemic at a low level although the progressive release of the stringency did not produce a clear increase of the contact number. The arrival of the omicron variant is clearly detected and characterised by a quick increase of interpeople contact, for most of the African countries considered in the analysis.

## Author summary

In this study, we show (1) that two main factors can explain the lower numbers of cases and deaths per inhabitants in Africa: an underestimation (by a factor 8.5) of the reported cases and deaths which directly results from the under capacities of the health systems at country level, but also a genuine effect by a factor 5.1 directly resulting from the smaller fraction of elderly people. We demonstrate (2) that the dynamics of the epidemic can be approximated deterministically by few variables only. Its time evolution is however highly sensitive to the initial conditions which makes it unpredictable at long term. Moreover, dynamics can largely vary from one country to another. For one country (Ghana), it is shown that very different epidemiological evolution can occur under strictly identical sanitary conditions. Finally, we reveal (3) that the impact of the control measures on the contact number is effective at short term and enabled to maintain the epidemic at a relatively low level, but it is more difficult to identify distinctly the long-term role of mitigation strategy. The omicron variant is very clearly detected in the recent evolution of the epidemic.

## Introduction

Africa has been the subject of relatively less attention in comparison to the other continents since the beginning of the pandemic of COVID-19. One reason for that is the apparent lower magnitude of the epidemic in contrast to what was primarily expected and in comparison to most of the other countries. At present, its specificities remain puzzling and numerous hypotheses have been made to explain this slower propagation [1–5], among which, (1) the relatively good preparation of the African countries after their notable experience of recent emerging epidemics; (2) the demographic age structure characterised by a lower proportion of aged people and a lower population density; (3) the climatic conditions which may foster or hinder the propagation of the disease; (4) a possible pre-existing partial immunity related to the exposure to other zoonotic coronaviruses or a reduced susceptibility to severe forms of the disease together with a specific role of comorbidities [5, 6]; Finally (5) an underascertainment of cases and deaths occurrences [5–8] due to insufficient diagnostic facilities, poorly adapted serological tests to detect the asymptomatic cases (in Africa, these tests give higher seroprevalence than expected which may result from cross-reactions of the test with other viruses and parasites in circulation on the African subcontinent), absence of registration and under-sampling. Although all these factors may have played a significant role, their quantitative influence remains unclear. In terms of total number of cases per inhabitant for instance, the difference with countries of other continents, in particular with Europe, appears considerable (by a factor around 10.9 on average for the cases and 8.4 for the deaths).

The questions about dynamics and dynamical complexity are important issues in epidemiology. The dynamics of epidemics is rarely simple. On the contrary, it is often highly unpredictable—sometimes even at very short term as it is the case for other diseases such as the Ebola Virus Disease [9]—until the propagation of the disease can be completely contained. Most of the epidemiological models can only produce very basic dynamical behaviours (often a single oscillation before converging to a stable situation, or a succession of strictly periodic oscillations) in comparison to the high complexity of the oscillations actually observed. This is an important limitation for epidemiological modelling. For this reason, to detect chaotic behaviours has been expected in epidemiology since the early 1980s. It was proven possible to generate more complex simulations with theoretical epidemiological models by either applying a periodical forcing on models of Susceptible-Exposed-Infected structure [10–12] or by combining predator-prey and Susceptible-Infected models [13, 14].

The possibility to extract chaotic models directly from epidemiological data is more recent [9, 15–17]. To allow valuable analyses of poorly predictable systems, the modelling approach should not use predefined model structure (e.g. models such as the SEIR models have a fixed structure and cannot produce complex dynamics; therefore, they cannot be used to detect chaos, neither to study the dynamics), and should make it possible to overcome the problem of sensitivity to the initial conditions. Based on chaos theory [18, 19], the global modelling technique [20] was designed for this purpose. A chaotic dynamics is defined by two main properties: determinism and high sensitivity to the initial conditions. The initial conditions, here, do not restrictively refer to the conditions at the very beginning of the outbreak. It refers to any initial conditions, be it taken at the earlier origin with the patient zero, after several days or weeks, or once the epidemic has reached its permanent dynamics. In a chaotic system, for a small perturbation, this high sensitivity will result in the exponential divergence of the trajectories, whenever this perturbation will be applied. The problem of modelling (hypothetically) chaotic dynamics can be stated as follows: If small changes in the initial conditions can give rise to completely different time evolution, then the modelling approach should not only enable to reproduce the single time evolution observed in practice, it should retrieve a set of equations able to simulate any of the time evolution made possible by the dynamics. To do so, the global modelling technique takes advantage of the state space (or phase space), an oriented space able to represent—all—the possible states of a given deterministic system. For this reason, this space is independent from the initial conditions. Moreover, as proven by the embedding theorems [21, 22], this state can be reconstructed from observational time series, establishing a powerful bridge between theory and applications.

Thanks to this bridge, the global modelling technique can be used to model chaotic dynamics directly from observational time series [23]; It can also be used to detect directional couplings under chaotic regimes [24] and to obtain interpretable sets of chaotic equations [15] without strong hypotheses. For COVID-19 in China, the approach enabled to obtain a model (**M**_2_) characterised by intermittency [17], revealing that, despite a control of high stringency put in place to achieve the zero-COVID strategy, the equilibrium was unstable and restarts were to be expected after an undetermined time. Facilitated by the emergence of the omicron variant, such a situation was confirmed almost two years later by a restart that broke out by the beginning of 2022. Such a restart appears fully expected now, but it was not at all at the time this model was obtained (06 April 2020). Of course, the approach can also be applied under non chaotic conditions.

Face to emerging or re-emerging diseases, in particular under a pandemic context, the question of the efficacy of the mitigation strategy is of first importance. This question has been investigated using different approaches. For the epidemic of COVID-19, most of the studies on the impact of mitigation strategies have been prospective and based on scenarios. Scenarios can help in determining the measures to be fostered. However, their ability to assess the genuine impact of the intervention policies highly depends on the hypotheses the scenarios have been built for (this limitation is not specific to epidemic scenarios [25]). Therefore, other approaches should be preferred to make a diagnostic of their impact *a posteriori*.

Day-by-day estimates of the effective reproduction number (the average number of secondary cases contaminated at time *t* by an infectious individual) is commonly used to track the evolution of epidemics. Various techniques were developed for this purpose [26]. However, the main aim of this number is to determine if the epidemic is on either growing or decreasing stage, but it cannot distinguish the effect of pharmaceutic versus non-pharmaceutic interventions. Therefore, it is not adapted to estimate the impact of control measures.

Some approaches have been developed to estimate the impact of non-pharmaceutical interventions, either by trying to separate the effect of less/more restrictive non-pharmaceutical interventions according to the growth rate [27], or by analysing the reproduction number in relation to the physical distancing and other control measures [28], or by reconstructing the infection rate functions [29] in SEIR models.

The aim of the alternative approach introduced in the present work is to reconstruct *β*(*t*) the time evolution of the average contact number directly from the daily evolution of newly infected cases. Such a reconstruction can be of particular interest to understand the efficacy of the mitigation strategy since one main role of the non-pharmaceutical strategies is precisely to reduce the interindividual contacts.

The purpose of the present study is to provide an overall perspective on the epidemic of COVID-19 in Africa since it broke out. Three main objectives are considered. The first one is to understand the low numbers of cases and deaths due to COVID-19 in Africa in comparison to the other continents, and their geographical variability at intracontinent scale. The second objective is to investigate the dynamics of the epidemics in Africa at the country scale, to explain its low predictability and to investigate its intercountry variability. The third objective is to assess the impact of the mitigation strategies by the reconstruction of the average contact number at the country scale from the earlier beginning of the outbreak.

## Materials and methods

### Databases

Six databases have been used in the analyses. (i) The *United Nations* [30] and (ii) the *World Bank* [31] databases are used to investigate some possible factors to explain the huge differences observed between Africa and other countries in the worlds—in particular in Europe—and among African countries in terms of cases and deaths of COVID-19. Two main variables are considered in the study: the proportion of aged people (≥ 60 years old) and the number of hospital beds per capita, the other variables (the poverty rate, the gross domestic product, the population density, the number of health workers, and the average number of doctors per inhabitant) having been rejected as factors of second order at continent scale.

Epidemiologic data are taken from (iii) the *Center for System Science and Engineering* of the *John Hopkins University* [32]. Three variables are used for each country: the daily number of new cases, the daily number of deaths and the daily number of recovering people. Note that cases and deaths counts include confirmed but also probable ones (when reported). Analyses are performed using the global modelling technique presented in the present section and discussed in the main manuscript section “Dynamical complexity and diversity”. A correction factor was applied to the time series in order to account for the underestimation of the health system. This factor, based on the number of hospital beds per capita will be introduced in the section “Geographical differences”.

Vaccination data used in this study are taken from (iv) *Our World in Data* [33]. This data set is updated regularly and provides, among others, the total number of vaccines administrated for 169 countries in the world with a daily time step.

The stringency index *i*_*ox*_ of (v) the *Oxford COVID-19 Government Response Tracker* (OxCGRT) dataset [34] is also used for comparison with our results in the section “Mitigation strategy impact”. The aim of this dataset is to address the need for continuously updated, readily usable and comparable information on policy measures from 1 January 2020. The stringency index focuses on containment and closure policies. It accounts for (1) school (2) workplace and (3) public transport closing, (4) public events cancelation, (5) stay-at-home requirements, restrictions on gathering size (6) internal movements and (7) international travel, and on (8) public information campaign. Each of these indicators is associated with a specific weight to compose the stringency index. Their individual contribution is set to zero when values are missing. The resulting index *i*_*ox*_ varies from 0 (no stringency) to 100 (maximum stringency). Since higher levels of stringency should be associated to lower numbers of contacts, the index of reverse variations defined as 100—*i*_*ox*_ will be used in the present study.

The residential duration and the retail-recreation time were taken from (vi) the *Google COVID-19 Community Mobility Reports* [35], it provides information from the 17 February 2020. Although some tips are made available to help the interpretation of these indices, the methodology used to generate the product is not provided with full details. Nonetheless, albeit rough, it brings some information that was found helpful for interpreting our results. All the data are expressed in comparison to a baseline day assumed to represent a normal value for that day of the week. This baseline day is taken equal to the median value estimated on the period 3 January 2020 to 6 February 2020 [35]. Two indices are used in this study, both provided with a 7-day moving average smoothing. The residential duration index *i*_*RD*_ is provided as a positive (negative) percentage when people will spend more (less) time at home than during the baseline period. An offset of 100% was added to this index, such as 100 + *i*_*RD*_, to facilitate the comparisons with the other variables. The retail and recreation index *i*_*RR*_ aims to measure the changes in total visitors at a country scale. It accounts for various categories of places including restaurants, cafés, shopping centres, theme parks, museums, libraries and movie theatres. Similarly, an offset was applied to this index, such as 100 + *i*_*RR*_, the 100% level corresponding to the reference situation.

### Countries of study

One hundred and three countries are considered in the whole world to identify potential factors that could explain the differences observed in the values of COVID-19 total cases and deaths at countries and continents scales: 47 in Africa, 43 in Asia, 36 in Europe and 24 in America. Their complete list is provided in S1 File. Only the countries of more than one million inhabitants were studied. Countries for which information was missing in the data bases (i) and (ii) (see previous subsection) were also removed. More specifically for Africa, South Sudan was not retained in the analysis due to missing value for the number of hospital beds per capita, Tanzania was also removed due to the government decision to no longer count COVID-19 cases and deaths since June 2020. For Gabon, the estimate of the number of bed per inhabitant made available in 2010 was found inconsistent and replaced by an estimate made on 2008, more reliable.

Time series from data base (iii) were used for the modelling study. Seventeen African countries were selected for this purpose. Sixteen of these are those with the most data available (no gaps longer than 2–3 days in the daily counts, and significant number of cases/deaths), while keeping some diversity in epidemiological dynamics observed. The last one (Togo), is a country with a limited amount of information that was kept in order to test the effectiveness of the data pre-processing.

### Time series pre-processing

A careful pre-processing is required to apply the global modelling technique. This was applied only to the time series from the John Hopkins database (cases and deaths), since data from the other sources were used only for preliminary statistics (annual values) or comparative analyses (external indices), not for modelling.

The following steps were applied to the time series: (1) information about anomalies were gathered for each country and complemented by a visual inspection of each studied time series to detect possible non reported anomalies, these anomalies were corrected manually when possible; Once these corrections applied (2) the cumulated time series (cases or deaths) were subsampled at a weekly time sampling leading to an ensemble of seven time series, each starting from a different day of week; (3) These seven time series were resampled at a hourly sampling using cubic splines. To keep as much as possible of the information of the very last days, the time sampling was increased during the very last week with a maximum gap of three days allowed (it was avoided to use the same last values for the seven time series in order to keep information about the dispersion, up to the very end of the studied period); (4) The average and standard deviation of the seven time series was computed to obtain an average estimate of the time evolution and of the error (deduced from the dispersion); (5) A geographical correction factor was then applied to the whole set of time series based on the efficacy of the health system in the country to account for underreporting. Finally (6) a Savitzky-Golai algorithm was used with a ±1 day window (a larger window of ±6 days was used to assess the contact number) to estimate the successive time derivatives, required to apply the global modelling technique (note that this was also applied to both the average and the individual time series to have an estimate of the error associated with the derivatives).

### Basic statistics for inter-country comparison

Basic indices are introduced to investigate the influence of factors that may have contributed to the large differences observed in terms of numbers of infections and deaths per inhabitant.

Two weighting factors *w*_*A*_ and *w*_*B*_ are used to compare the numbers of cases and deaths at country scale. The former one is based on the proportion of aged people and defined as

$$\begin{array}{c} \frac{1}{w_{A}}=\frac{N_{\geq 60}}{N}, \end{array}$$ (1)

with *N*_≥60_ the number of inhabitants of 60 years old and over, and *N* the total number of inhabitants; The latter one is defined as

$$\begin{array}{c} \frac{1}{w_{B}}=\frac{n_{bed}}{n_{bed}^{ref}}, \end{array}$$ (2)

and based on *n*_*bed*_ the number of hospital beds per inhabitant and $n_{bed}^{ref}$ a reference number taken equal to 5.5 hospital beds per 1 000 inhabitants corresponding to the average value in European countries. The weighted numbers

$$\begin{array}{c} I_{TOT}^{w}=w\times I_{TOT}, \end{array}$$ (3)

and

$$\begin{array}{c} D_{TOT}^{w}=w\times D_{TOT}, \end{array}$$ (4)

were then estimated for each country considering the influences of the proportion of elderly people (*w* = *w*_*A*_), the number of hospital beds (*w* = *w*_*B*_) or both (*w* = *w*_*A*_ × *w*_*B*_) together. In the former case, the weighted numbers are given per inhabitant of 60 years old and more; In the second case, per inhabitant considering a number of hospital beds brought back to a situation corresponding to $n_{bed}^{ref}$; In the latter case, per inhabitant of 60 years old and more, and under hospital beds conditions brought back to the reference number $n_{bed}^{ref}$.

### The global modelling technique

In the second part of the analysis, a versatile modelling approach—the global modelling technique [24]—is used with the aim to characterize the epidemic of COVID-19 in terms of dynamical regime.

The global modelling technique used in the present study aims to obtain sets of governing equations directly from observational time series, without strong hypotheses [23, 36–38]. It was first applied in the 1990s to model chemistry reactions [39]. Being based on the theory of nonlinear dynamical systems, the present approach is particularly well suited to cope with epidemiological dynamics highly sensitive to the initial conditions as previously illustrated [9, 15, 17]. In contrast to most of the other data driven approaches, it can be applied to very small data sets (starting from a single time series), and even under scarce conditions (a few cycles, subsampled and noisy conditions) [24]. In its differential form, when a single time series is considered, the aim of the approach is to obtain a set of ordinary differential equations of canonical form

$$\left\{ \begin{array}{c} {\dot{X}}_{1}=X_{2}\\ {\dot{X}}_{2}=X_{3}\\ \;\;\;\;\;\;\vdots \\ {\dot{X}}_{n}=Q\left(X_{1},X_{2},..,X_{n}\right), \end{array}\right.$$ (5)

where the dots denote the first derivatives, *X*_1_ to *X*_*n*_ the successive derivatives of *X*_0_ the cumulative number of new cases or deaths (including *X*_1_ the daily number of new cases or deaths), *n* the model dimension, and *Q* a polynomial function which structure has to be retrieved in the modelling process. For the present analysis, the model dimension was fixed equal to *n* = 3, and the maximum polynomial degree to *q* = 3. Structure identification is a mandatory stage in the modelling process far from being trivial. Indeed, direct parameter identification without structure identification will lead, quasi systematically, to diverging models. The GPoM algorithm developed in R language by our team [40] was used to identify the model structure. Structure selection is operated in several stages. (i) The first selection stage is performed by ordering the monomials from smaller to higher importance (in terms of efficacy to reduce the residual signal resulting from the parameter identification). For a general formulation with *p* terms in the polynomial, this process enables to reduce the number of possible models from 2^*p*^ to (*p* + 1) models. This is a drastic selection. In the present situation (*n* = 3, *q* = 3), the full polynomial will have *p* = 35 terms. The present algorithm will then reduce the number of possible models in the basket from more than 3.4 ⋅ 10^10^ models to thirty-six models, only. (ii) The remaining candidates are then integrated numerically to test their robustness. Diverging models (presenting values larger than four times the standard deviation of the original variable) and inconsistent models (i.e. presenting negative numbers of cases or deaths) are systematically rejected. (iii) Considering their dynamics, remaining models are then classified, after the convergence is reached, as: fixed points, periodic cycles (of period 1, 2 or more), or as unclassified. Models presenting the higher consistency in terms of range are considered more realistic (fixed points are then rejected in the present situation; and periodic models of period 1 as well, if any model of higher complexity is also obtained). Finally (iv), the remaining models are then chosen in terms of forecasting performances. If several models presenting the same capacities remain at this stage, the model of smallest size is preferred. A detailed description of the algorithm, its application procedure, and its performances are provided in [24].

This algorithm has proven a very high level of performance, enabling to retrieve original sets of equations among tremendous number of possible ones. Its robustness was successfuly tested under various types of degraded conditions (short time series, undersampling, noise, etc.). It could be applied to very diversified realms (including epidemiology, eco-epidemiology, but also soil eco-hydrology, hydro-geology, agronomy). At present, most of the chaotic models directly obtained from environmental time series were obtained with this algorithm.

Models validation was performed according to the forecasting performances, estimated on an independent window using the error growth defined as

$$\begin{array}{c} |e_{\tau }\left|=\right|{\hat{X}}_{t}\left(\tau \right)-X_{t+\tau }^{obs}|, \end{array}$$ (6)

where ${\hat{X}}_{t}\left(\tau \right)$ denotes the model forecast performed at time *t* for a prediction time *τ*, and $X_{t+\tau }^{obs}$ the observed value (of cases or deaths per million inhabitants) at time *t* + *τ* (corresponding to the forecasts time). The forecasting performances are summarized in S2 Table based on the percentage of error of the number of new daily cases/deaths per million inhabitants at a 10-day prediction horizon (with a 90% confidence level).

### Estimating the mitigation strategy impact

In the third part of the analysis, an approach is introduced and used to estimate the time evolution of the contact number, directly from observational time series.

Assuming that the people exposed to infected cases become infectious after a time delay *τ*, the continuous variations of the average number of contact *β* per person and per day can be estimated as

$$\begin{array}{c} \hat{\beta }\left(t\right)=\upsilon \frac{NI_{t}^{\left(1\right)}}{\left(S_{t=0}-I_{t}^{\left(0\right)}-V_{t}\right)i_{t-\tau }} \end{array}$$ (7)

with *N* the population size, $I_{t}^{\left(1\right)}$ the daily number of new cases at time *t*, *S*_*t* = 0_ the initial number of susceptible people (taken equal to *N*), $I_{t}^{\left(0\right)}$ the cumulated number of infected people since the beginning of the epidemic at time *t*, *V*_*t*_ the number of vaccinated people at time *t*, *i*_*t*−*τ*_ the total number of infectious people at time (*t* − *τ*), and *υ* a correction coefficient close to 1 estimated empirically. Vaccination has to be taken into account here because it will reduce the number of susceptible people which role is important here. Estimates of the recovering ratio, morbidity ratio and effective reproduction number **R**_*t*_ can also be derived from it. A complete introduction of the approach is presented in S1 Appendix.

To check its validity, the method was tested on a complicated version of compartment model including both symptomatic and asymptomatic compartments, and run under an exogenous social forcing of weekly period characterised by differentiated specifications for the symptomatic and asymptomatic compartments. Two application scenarios of the approach are presented in S2 Appendix to illustrate its efficacy. These simulations clearly point out the very different behaviour of the contact number compared to the reproduction number: it shows that once the vaccination has reached a sufficient proportion of population, the contact number can be increased to its original level keeping the outbreak under control (**R**_*t*_ < 1); and that the methodology here developped can be efficiently used to reconstruct the time variations of both *β*(*t*) and **R**_*t*_.

## Results

### Geographical differences

Since the beginning of the pandemic of COVID-19, the total numbers of cases and deaths per capita exhibit huge geographical differences not only from one continent to another but also between the countries within each continent.

For Africa, two factors of first order have been identified to explain these geographical differences. The proportion of elderly people is the first factor. Indeed, as most of the cases of COVID-19 requiring hospitalization, or, leading to death, concern elderly people, demography is expected to play a key role. In Africa the proportion of elderly people (≥ 60 years old) ranges from 3% to 6% for most of the countries, it is a bit higher in the Southern part of Africa (7%-9%) and in North Africa (7%-13%). In contrast, it is significantly higher for instance in Europe where it ranges from 20% to 30% (25% on average).

The second factor is linked to the health systems capacities. The ability to track the evolution of an epidemic over time necessarily relies on the capacities of the health system at the country scale. The number of hospital beds per capita can be a good proxy of this capacity in particular for the analysis of the total number of cases and deaths. Capacities and efficacy of the health systems are highly variable from one country to another. In Europe, for instance, the number of hospital beds per inhabitant is close to 5.5‰ on average with variations ranging from 2.1‰ to 8‰. In Africa, these values are systematically lower. The highest values are found in the Northern part (up to 3.2‰ in Lybia) and Southern part (up to 2.7‰ in Namibia) of Africa. Capacities are lower than 1‰ in most of the African countries, and can reach extremely low values in some of them (0.4‰ in Mauritania, Burkina-Faso, Chad and Côte d’Ivoire, 0.33‰ in Ethiopia, 0.3‰ in Senegal and Guinea, 0.2‰ in Madagascar, down to 0.1‰ in Mali).

Intracontinent contrasts in the total numbers of cases and deaths are particularly marked in Africa where the coefficient of variation ($c_{v}^{0}=\sigma /\mu$ in Tables 1 and 2) reaches 1.63 for the cases and 1.82 for the deaths, in comparison to the other continents (in the ranges [0.39; 1.13] and [0.52; 1.09], respectively). Considering the weighted numbers of cases or deaths, using *w*_*A*_ to account for the proportion of elderly people, or *w*_*B*_ for the health system capacities (see section Material and methods for details), enables to reduce considerably the inter-country contrasts on the African continent. The contrast reduction relating to health systems capacity is particulary noticeable in Africa, especially for the deaths ($c_{v}^{B}/c_{v}^{0}=0.70$ for cases and 0.54 for deaths). The reduction relating to elderly people is also clear ($c_{v}^{A}/c_{v}^{0}=0.91$ for cases and 0.79 for deaths). Moreover their combined effect (*w* = *w*_*A*_ × *w*_*B*_) confirms the role of these two factors ($c_{v}/c_{v}^{0}=0.59$ for cases and 0.54 for deaths).

**Table 1:** Statistics of *I*_*TOT*_ the total number of COVID-19 cases per thousand inhabitants on 10 January 2022 since the beginning of the pandemic. Four variables are considered: the unweighted number, the numbers weighted by the proportion of aged people (*w*_*A*_), by the number of hospital beds per inhabitant (*w*_*B*_) or both (*w*). The coefficient of variation *c*_*v*_ = *σ*/*μ* is calculated for each variable (with *σ* the standard deviation and *μ* the mean). The variation coefficients of the weighted numbers are then compared to the unweighted ones. Statistics are provided five ensembles (Europe, America, Eastern and Southern Asia and Africa), and for the whole world.

Cases	*I*_*TOT*_			$I_{TOT}^{w_{A}}$				$I_{TOT}^{w_{B}}$				$I_{TOT}^{w}$			
Region	*μ*	*σ*	$c_{v}^{0}$	*μ*	*σ*	$c_{v}^{A}$	$c_{v}^{A}/c_{v}^{0}$	*μ*	*σ*	$c_{v}^{B}$	$c_{v}^{B}/c_{v}^{0}$	*μ*	*σ*	*c*_*v*_	$c_{v}/c_{v}^{0}$
Europe	146.50	56.88	0.39	582.6	235.9	0.40	1.04	187.8	106.6	0.57	1.46	751.6	452.3	0.60	1.55
America	70.28	47.86	0.68	491.2	275.0	0.56	0.82	227.8	145.0	0.64	0.93	1873.4	1566.1	0.84	1.23
Eastern Asia	26.43	29.88	1.13	251.0	403.4	1.61	1.42	54.2	42.5	0.78	0.69	523.2	484.6	0.93	0.82
Southern Asia	67.05	63.14	0.94	830.3	863.4	1.04	1.10	198.1	169.3	0.85	0.91	2862.7	3321.4	1.16	1.23
Africa	13.39	21.85	1.63	207.9	309.1	1.49	0.91	51.1	58.4	1.14	0.70	878.6	850.3	0.97	0.59
World	65.76	68.31	1.04	463.7	508.5	1.10	1.06	139.9	134.2	0.96	0.92	1342.1	1835.9	1.37	1.32

**Table 2:** Same as Table 1 for *D*_*TOT*_ the total number of COVID-19 deaths per thousand inhabitants since the beginning of the pandemic.

Deaths	*D*_*TOT*_			$D_{TOT}^{w_{A}}$				$D_{TOT}^{w_{B}}$				$D_{TOT}^{w}$			
Region	*μ*	*σ*	$c_{v}^{0}$	*μ*	*σ*	$c_{v}^{A}$	$c_{v}^{A}/c_{v}^{0}$	*μ*	*σ*	$c_{v}^{B}$	$c_{v}^{B}/c_{v}^{0}$	*μ*	*σ*	*c*_*v*_	$c_{v}/c_{v}^{0}$
Europe	2.17	1.14	0.52	34.76	22.08	0.63	1.21	10.01	5.73	0.57	1.09	40.51	26.17	0.65	1.23
America	1.67	1.30	0.78	104.54	92.81	0.89	1.14	52.91	53.66	1.01	1.30	500.79	623.97	1.25	1.59
Eastern Asia	0.23	0.19	0.82	26.41	33.75	1.28	1.56	7.18	8.91	1.24	1.52	81.34	105.17	1.29	1.58
Southern Asia	0.77	0.84	1.09	107.42	107.10	1.00	0.92	28.08	23.82	0.85	0.78	438.34	476.44	1.09	1.00
Africa	0.26	0.47	1.82	64.85	93.22	1.44	0.79	16.02	15.78	0.98	0.54	304.91	299.40	0.98	0.54
World	1.03	1.19	1.15	68.08	85.23	1.25	1.09	21.85	29.47	1.35	1.18	275.35	400.84	1.46	1.27

All the other factors tested in the analysis were not able to explain the geographical differences, since leading to increase the contrast observed between the continents or between the countries, or to maintain it close to one ($c_{v}^{\;.}/c_{v}^{0}\;≳\;1.$).

Demographic and health system factors do not have such a systematic effect to reduce the contrasts on the other continents. Obviously, it is efficient at the global scale only to reduce the contrasts observed in the number of cases when taking the health system capacities into account (for which $c_{v}^{B}/c_{v}^{0}$ reaches 0.92 on average). Note that the effect of this factor in Europe ($c_{v}^{B}/c_{v}^{0}=1.46$) is in opposition to the other parts of the world (in the range [0.69; 0.93]), however, the contrast among the European countries being relatively much lower ($c_{v}^{0}=0.39$) this increased dispersion still remains marginal after applying the weighting (contrast between the countries is actually the lowest in Europe for all the situations, whatever weighting is applied—or not—and for both cases and deaths).

Contrast reduction is far not systematic in all the other situations. On average, results show that the contribution of elderly people is significant only for the cases in America ($c_{v}^{A}/c_{v}^{0}=0.82$) and for both the cases and deaths in Southern Asia ($c_{v}^{A}/c_{v}^{0}=0.92$ and $c_{v}^{B}/c_{v}^{0}=0.78$). Elsewhere, it is not.

**Fig 1:**
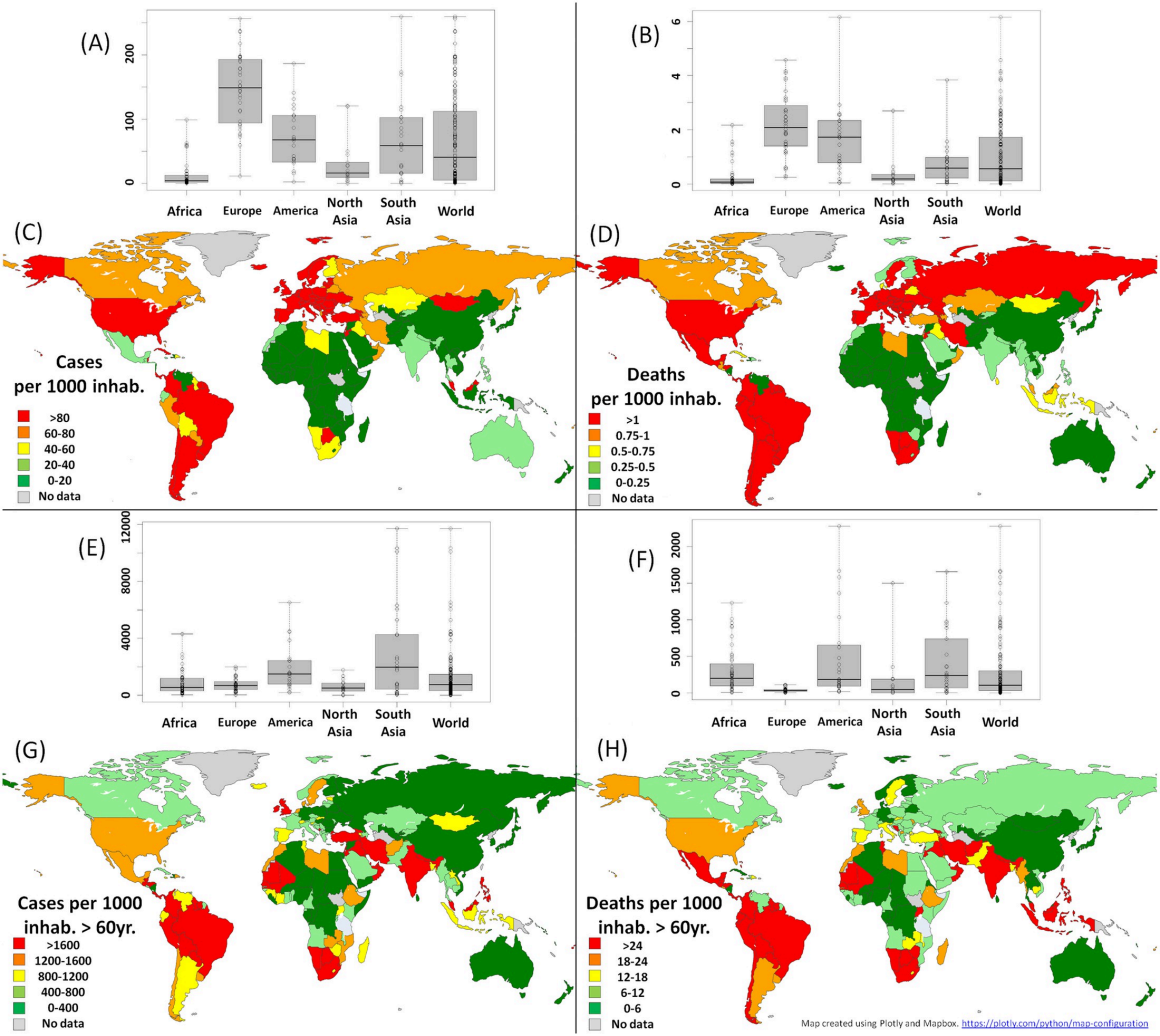
Statistical and geographical distributions of COVID-19 in the world. Boxplots and geography of the numbers of COVID-19 cases (A and C) and deaths (B and D) per thousand inhabitants before health system correction is applied, and of the numbers of COVID-19 cases (E and G) and deaths (F and H) per thousand inhabitants of 60 years old and over, with health system correction based on the number of hospital beds per inhabitant applied. Geographical distributions resulting from these two factors separately are presented in S1 Fig. Results are presented for each continent separately (boxplots) and for the whole world (maps). Only countries with more than 1 million inhabitants are considered in the boxplots analysis where the rectangles correspond to the 25% and 75% centiles, and the extreme values to the minimum and maximum of the distributions; the central bar within the box corresponds to the median. Map created using Plotly and Mapbox. https://plotly.com/python/map-configuration.

Despite their limited effect at the global scale, these two factors clearly enable to reduce the dispersion of the numbers of cases and deaths between Africa and the other continents. The contrast is particularly flagrant between Africa (with *I*_*TOT*_ = 13.4 cases and *D*_*TOT*_ = 0.26 deaths, per thousand inhabitants on average) and the whole world (65.8 and 1.03, respectively) before any weighting is applied (see Fig 1A–1D); It reduces considerably after accounting for the two factors ($I_{TOT}^{w}=878.6$ and $D_{TOT}^{w}=304.9$ in Africa against $I_{TOT}^{w}=1342.1$ and $D_{TOT}^{w}=275.3$ for the whole world, each per thousand inhabitants of 60 years old and over, Fig 1E–1H). The contrast is thus reduced by 3.2 for the cases and 3.6 for the deaths.

The demongraphic factor enables a reduction of contrast in comparison to the whole world by a factor 2.2 for the cases, and 3.8 for the deaths; and the health system by a factor of 1.8 for the cases and 2.9 for the deaths (and even by 2.8 and 5.2 with Europe, respectively).

Geographically, using the weighting factor *w*_*B*_ as a proxy of the under-ascertainment, underreporting is estimated to be moderately high in the Northern part (Lybia, Tunisia and Algeria are in the range [1.7; 2.9]), Southern part (Namibia, South Africa, Eswatini, Zambia, Botswana and Zimbabwe, in the range [2.0; 3.2]) Eastern (Rwanda, 3.4) and Central part of Africa (Equatorial Guinea and Congo, in the range [2.6; 3.4]). Contrarily, it is estimated particularly high, for the most in West Africa (Burkina Faso, Chad, Mauritania, Niger, Côte d’Ivoire, Guinea, Senegal, in the range [13.7; 18.3], and up to 55. for Mali), and Estearn Africa (16.7 for Ethiopia). See S1 File for details.

These two factors being taken into account (that is, both the under-ascertainment of the cases/deaths and the proportion of elderly people), the analyses reveal that the total number of cases in Africa (878.6 cases per inhabitant of 60 years old and older) is actualy only slightly higher than what is observed in Europe (751.6). It is significantly (1.5 times) lower than the global average, the highest values being found in Southern Asia (2862.7) and America (1873.4), the lowest ones in Eastern Asia (523.2). Concerning the deaths, the number (304.9 deaths per inhabitant of 60 years old and older) is moderately higher in Africa (1.1 times) than at global scale (275.3), significantly lower than America (500.8) and Southern Asia (438.3), but much higher than Eastern Asia (81.3) and Europe (40.5).

When taking into account these two factors, the geographical map highlights (see Fig 1C and 1D before, and Fig 1G and 1H after) particular geographical contrasts in Africa, in the southern part of the continent (mainly in Namibia, South Africa, Botswana, Swaziland and Zimbabwe), in West Africa (Mali, Mauritania and Senegal), and some more local behaviours (in Ouganda and Tunisia). It also reveals a high level of cases (in the range [1200; 1600]) and/or deaths (in the range [12; 24], both per inhabitant of 60 years old and over) in Morocco, Lybia, Zambia, Malawi, Mozambique and Madagascar.

The maps of the separate contribution of the two factors show that the capacity of the health system enables to explain only moderatly the values observed in West Africa (Mauritania, Mali and Senegal), in particular regarding the deaths (see S1(B) Fig). The map also reveals a specific behaviour for South Africa and neighbouring countries where higher levels of cases and deaths are associated with comparatively higher proportion of elderly people, even when taking their proportion into account.

### Dynamical complexity and diversity

**Fig 2:**
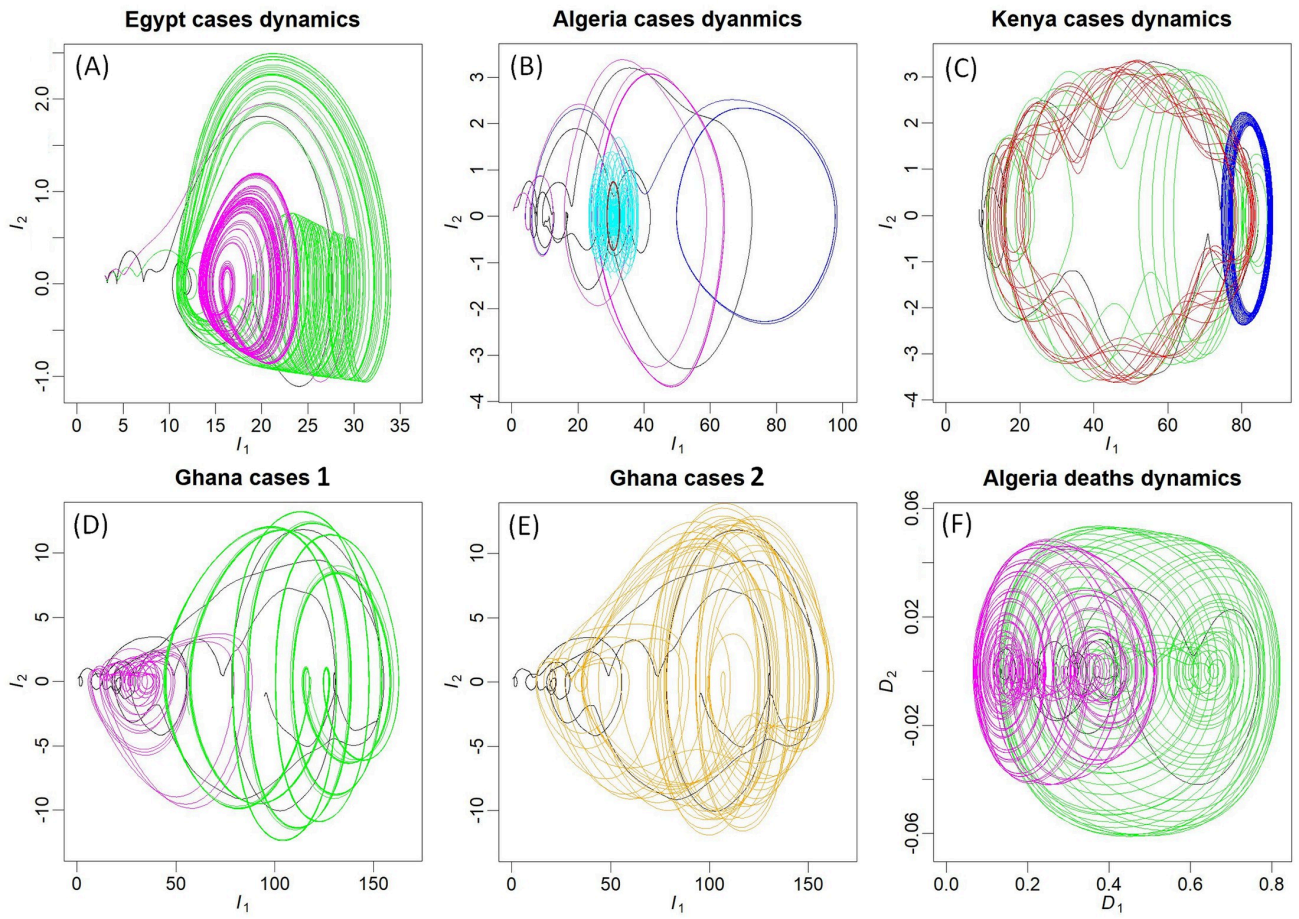
Attractors (differential phase portraits in (*I*_1_, *I*_2_) projection) obtained by tuning the models parameters (or from alternative models) for cases or deaths dynamics of four countries. In each case, the original phase portrait is also reported (in black line) for comparison. The two chaotic attractors presented in (A) for the cases dynamics in Egypt were obtained from Eq. (3) in S3 Appendix with *κ* = 1.25 (in magenta) and *κ* = 1.7 (in green). The three non chaotic attractors presented in (B) for the dynamics of cases in Algeria were obtained from Eq. (1) in S3 Appendix with (*κ*_1_, *κ*_2_) = (1., 0.75) (in magenta), (*κ*_1_, *κ*_2_) = (1., 1.2) (in blue), and (*κ*_1_, *κ*_2_) = (1.7, 1.) (in brown, with its transient in cyan). The attractors for the dynamics of cases in Kenya (C) were obtained from Eq. (6) in S3 Appendix with *κ*_1_ = *κ*_2_ = 1. (in green), (*κ*_1_, *κ*_2_) = (1.08, 0.9901) (in red), (*κ*_1_, *κ*_2_) = (1, 0.9882353) (in blue). The two attractors (in magenta and green) presented for cases dynamics in Ghana (D) were both obtained from equations (5) in S3 Appendix with *κ* = 0.85, but from different initial conditions revealing to a situation of bistability; The attractor presented in (E) was obtained from Eq. (13) in S3 Appendix, presenting one term less than Eq. (5). The two chaotic attractors presented in (F) for the dynamics of deaths in Algeria were obtained from Eq. (14) (in green) and (23) (in magenta), both in S3 Appendix, and correspond to two different epidemiological situations.

The global modelling technique was applied to seventeen countries (see section Material and methods for details). The time series used for this purpose are presented in S2 and S3 Figs. Models were obtained for eleven of the selected countries for the dynamics of COVID-19 cases, and for nine of them for the dynamics of the deaths. Several of them are presented in Fig 2 as phase portraits to illustrate the diversity of dynamical behaviours; the other ones are provided in S4 and S5 Figs. Phase portraits are projections of the state space. This space provides a geometrical representation of the dynamics. Under proper conditions of observability [41], this space can be reconstructed [22] from a single observational variable. This space being independent to the initial conditions (since it contains, in principle, all the initial conditions), it can be used to obtain models for chaotic dynamics and is well suited to tackle with epidemiological behaviours which long-term dynamics is often poorly predictable. Not to require any strong assumption about the underlying processes at work is another interest of this modelling approach. It can thus be used to reproduce the original dynamics as a whole in a compact formulation [24], including processes that are not explicitly described, difficult to quantify, or even possibly unknown. The equations of the models obtained in the present study are all provided in S3 Appendix, together with the initial conditions used in the simulations. The dynamical regimes reached by these models are summarized in S1 Table. Their validation was carried out based on their forecasting performances (see S6 and S7 Figs and S2 Table) by the end of 2021. Their application to the most recent evolution of the epidemic, after the emergence of the omicron variant, revealed the high non stationarity of the dynamics resulting from this mutation.

Chaotic regimes (metastable in some cases) were obtained with ten models: six ones for the cases dynamics, five for the deaths. By definition, chaos refers to dynamics that are—simultaneously – deterministic and unpredictable [18]. Detecting chaos from observational data requires—as a necessary condition—to detect determinism in the first place [42]. For this reason, the global modelling technique is particularly well designed to detect chaos [19]. An in-depth analysis of the models enabled to confirm rigorously the chaotic properties when it was the case. The multiple monotonic branches in first return maps were used for this purpose S8 and S9 Figs; these reveal the presence of folding in the models state space and are obvious indicators of chaotic dynamics.

It is sometimes believed that chaotic models should be avoided because they produce dynamics unpredictable at long term [43]. This idea results from a misunderstanding. To perform optimal predictions of an observed behaviour, a model should be as close as possible to the original dynamics. If the original dynamics is chaotic, ideally, the model should also be chaotic. Using a non chaotic model for performing predictions of a chaotic system will also lead to a divergence between the model and the observation since the original dynamics is chaotic. Moreover, in such a situation, a non chaotic model will not be able to estimate the forecasting error resulting from the divergence of the dynamics. However, it is also true that the model dynamics should not be more unstable than the observed system.

Models of less interest were obtained in several cases (see S10 and S11 Figs). Their interest is regarded as poorer because their dynamics is obviously oversimplified in comparison to the original observations (these are converging either to a fixed point or to period-1 cycles, and no more complexity could be obtained from them). They were kept for their relatively good forecasting performances resulting from their long transient to reach the (trivial) attractor. These models are—local—rather than—global—models, since unable to capture the dynamics globally. No model could be obtained for Morocco, Togo, Zimbabwe and Zambia.

Small modifications of the parameter values were applied to the models to investigate the variations of the dynamics under such perturbations. In most of the cases, more developed chaos could be generated (e.g. for cases in Egypt, see Fig 2A) which is a very common situation in bifurcation diagrams. No complexity could be generated with some models (e.g. for the cases in Algeria), but their tuning enabled to get oscillations around very variable mean levels (Fig 2B). An important diversity of dynamics could be generated with other models (e.g. for the cases in Kenya), some characterised by very slow oscillations of large amplitude more or less altered (through parameter tuning) by quick oscillations of small amplitude; or by dynamics converging to a period-1 cycle of high mean level of new infection (Fig 2C).

**Fig 3:**
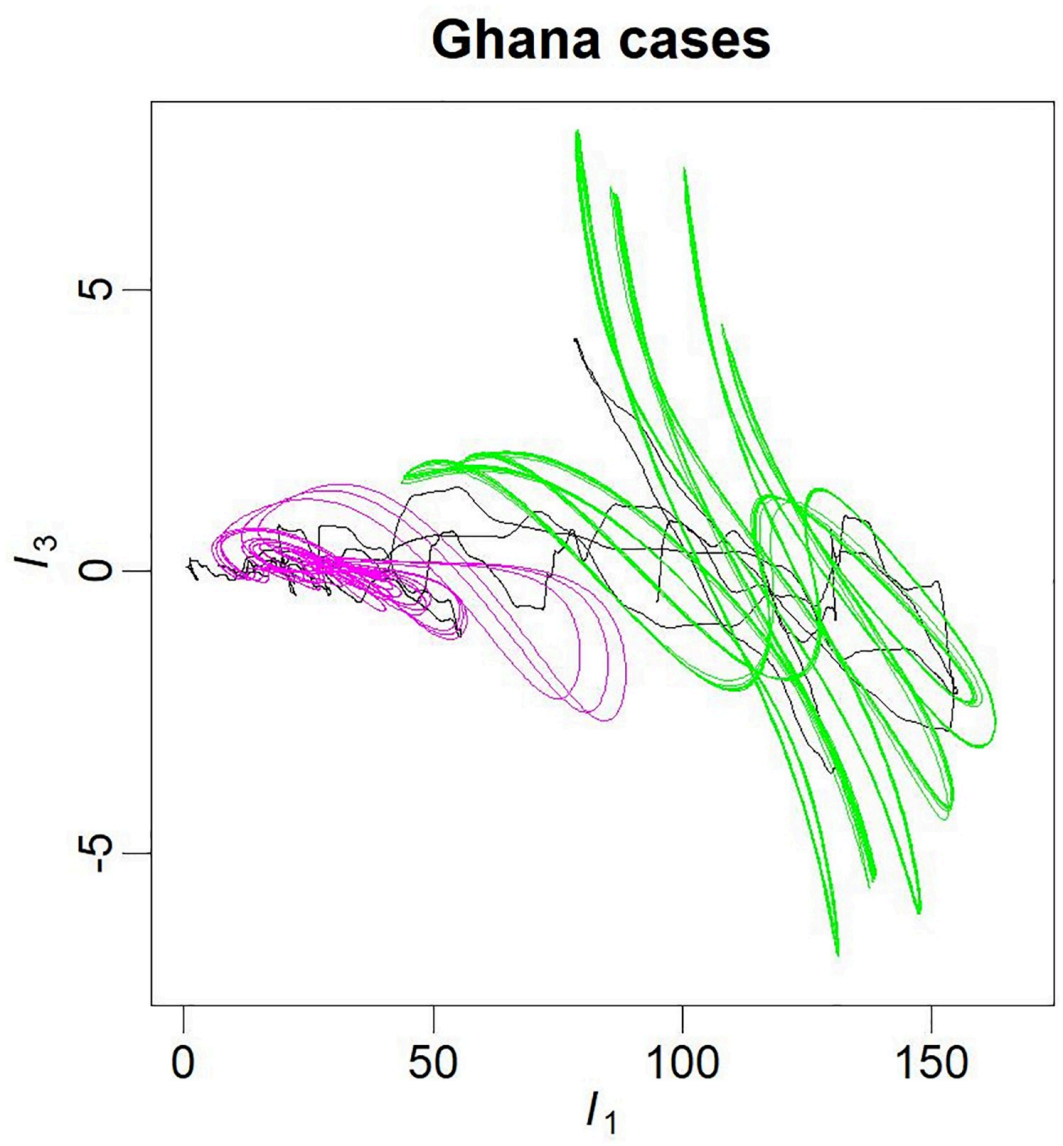
Differential phase portraits in (*I*_1_, *I*_3_) projection of the dynamics of daily new cases for Ghana. Observations (black lines) and model simulations (colored lines), are the same as the ones presented in Fig 2D.

A very unusual situation was found for the dynamics of cases in Ghana. A slight tuning of one of the parameters (*κ* = 0.85 in Eq. 5, see S3 Appendix) enabled to reach a situation of bistable chaos: two separated chaotic attractors (one of the two being very close to a period-8 cycle) corresponding to distinct attraction basin in the state space (see Fig 2D). Surprisingly, the two attractors present a large overlapping in their range of new infections per day, requiring a specific shape of their basins of attraction, as illustrated in Fig 3. Interestingly, a second model (Ghana-2) was also obtained from the same observations. This model gathers the two sub-basins in a single one, leading to a larger and much more complex attractor (see Fig 2E and S9 Fig).

Two models were also obtained for the deaths in Algeria. These are presented in Fig 2F after the transients were removed. Their dynamics correspond to very different epidemiological situations since the largest one is non chaotic and produces rapid increases of the number of infections whereas the smallest one is close to chaos but produces slow increases.

### Mitigation strategy impact

**Fig 4:**
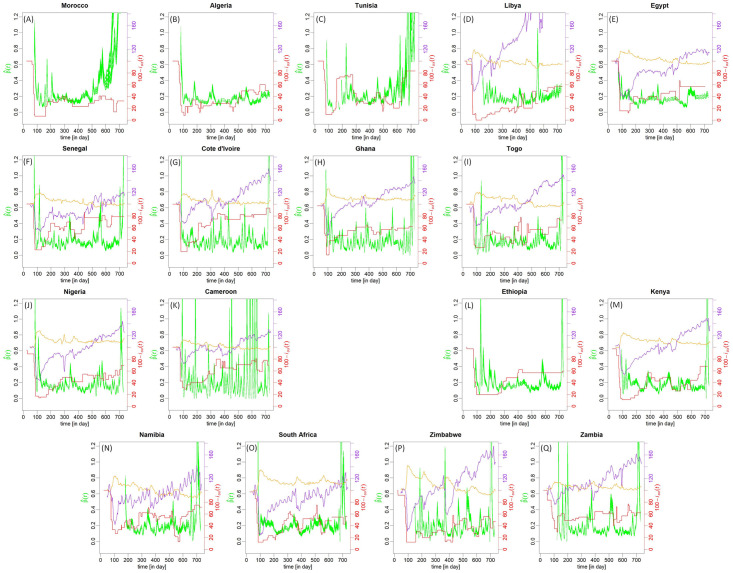
Average number of contact estimated per person per day. Average number of contact per person per day (green lines) reconstructed from the daily time series of new cases from 23 January 2020 (day 23) to 18 January 2022 (day 740) for seventeen countries (panels are organised geographically on the figure). For each country, an ensemble of 54 estimations is presented, obtained by varying the exposure duration (from 4.5 to 5.5 days), the sickness duration (from 4.5 to 5.5 days), the distribution shape (three types) and the percentage of asymptomatic (from 25% to 35%). The (reversed) index of stringency 100 − *i*_*ox*_(*t*) [in %] (red line), from the University of Oxford is also provided. When available, the percentage of residential duration (100 + *i*_*RD*_(*t*) in orange) and retail-recreation time (100 + *i*_*RR*_(*t*) in purple) are also plotted (in comparison to the median of the period 3 January to 6 February 2020 which represents 100%).

Estimates of the daily variations of the average number $\hat{\beta }\left(t\right)$ of contact per person and per day were performed for the seventeen countries selected in the study. These variations are presented in Fig 4. For most of the countries, a drastic decrease is clearly observed at the beginning of the epidemic down to a level close to 0.2 contact per person and per day. This decrease is followed by a slower decrease, sometimes associated with more or less complex oscillations, so that the minimum is reached after a delay of 50–100 days (e.g. Morocco and Côte d’Ivoire), or even more (up to 150 days or more) for some countries (e.g. Egypt, Kenya and South Africa). After this decrease, the signal is generally characterised by complex oscillations which amplitude highly varies from one country to another. Minimum values rarely go above 0.1. A quick increase is then observed in most of the cases by the beginning of 2022, coinciding with the arrival of the omicron variant. Very specific behaviours are observed for some countries. This is the case for Morocco for which a rapid increasing trend of the contact number is observed starting from the second part of 2021. A similar behaviour is observed in Tunisia, although the start is delayed toward the end of 2021. Cameroon exhibits stochastic-like variations of $\hat{\beta }\left(t\right)$ directly resulting from the irregular reporting of cases in the original time series (see S2(K) Fig).

The error associated with the estimates of the contact number are mostly due to the approximative knowledge of the length of infected period. The total error is generally lower than ±0.03 contact per person and per day. Larger levels of dispersion (± 0.15) were found only for Morroco and Tunisia by the end of 2021 / beginning of 2022.

The time evolution of the reverse stringency index (100—*i*_*ox*_) is also reported for comparison with the contact number. It shows a drastic decrease in the earlier development of the outbreak, followed by oscillations of varying amplitude, in time, and from one country to another. For most of the countries, these oscillations are associated with an increasing trend more or less marked from one country to another.

The time evolution of the indices of residential duration 100 + *i*_*RD*_(*t*) and retail and recreation 100 + *i*_*RR*_(*t*) are also reported, when available. The behaviour of the latter can also be described by a drastic decrease during the earlier spread of the disease, followed by (often) obvious increasing trend, agitated by small oscillations. It is the opposite for the former (sudden increase followed by a decreasing trend).

Despite the overall agreement (or reversed agreement) of the contact number with the three indices, the relationship appears more complex when considering the variations in more details.

## Discussion

### Underreporting and demographic effects

Two main factors were identified to explain the lower number of cases/deaths in Africa in comparison to the other continents. These two factors are very different in nature. On one hand, the capacities of the health systems reveal an important bias resulting from underreporting. Indeed, the presence of health care facilities is a condition to have the cases/deaths being reported. When no facilities are available, the cases/deaths will not be reported, leading to underreporting. The reference number of hospital beds per inhabitant being taken equal to 5.5‰, the analyses lead to an under-ascertainment by a factor 8.7 on average for the African continent (each country being considered independently to its population size). On the other hand, the age pyramid plays a direct role in the total numbers of cases and deaths. Its effect reflects a real situation: Indeed, even after accounting for the effect of underreporting, the numbers of cases and deaths remain significantly lower in Africa in comparison to the whole world (by a factor 2.7 for the cases and 1.4 for the deaths), and this directly results from the lower proportion of elderly people. This explains why, despite insufficient diagnostic capacities, health facilities have not been overwhelmed by patients.

Geographically, specific behaviours were observed in Mauritania, Mali and Senegal. These countries are characterised, at the same time, by—particularly low—health system capacities and proportion of elderly people. This may have led to over-estimate the correction in comparison to their neighbouring countries and finally, to over-estimate the number of cases and deaths when estimating the role of these two factors. Comparatively, most of the other countries in Southern and Northern African regions, are characterised by a relatively larger proportion of elderly people which may have contributed to a quicker development of the epidemic leading to high levels of cases and deaths, despite a higher capacity of the health system in comparison to the other African countries. Long-term care facilities for elderly people are much more developped in South Africa (and possibly in its neighbouring countries) than in the African continent in general [3]. This has very likely increased the communication of the disease and may thus explain the larger numbers of cases and deaths. It is interpreted here, at least for a part, as a real effect rather than a reporting bias.

### Chaotic dynamics

Low-dimensional chaotic models could be obtained for numerous countries, both for the dynamics of cases and deaths. This provides strong arguments for chaotic dynamics underlying the epidemics of COVID-19 in Africa. This detection is highly consistent with the model obtained for COVID-19 at the very beginning of the pandemic (05 February 2020) in China [17, 44]. To detect low-dimensional chaos from epidemiological time series is not only a challenging task. It is also an important issue as practical consequences follow: It shows that determinism plays a dominant role in the dynamics and that deterministic modelling makes sense. It also reveals that the unpredictability of the dynamics largely relies on the nonlinear (deterministic) couplings taking place between few main variables at play, rather than on a very high number of variables.

The large difference of performance observed between the models obtained for cases and deaths may be found surprising. Indeed, cases and deaths of COVID-19 are epidemiologically coupled, so that similar results may have been expected. Though, depending of the nature of this coupling, the coupled variables may exhibit very different behaviours. In some cases, such a dynamical coupling could be reconstructed algebraically from observational data at other geographical places (for COVID-19 in China [17] or for other diseases such as the Ebola Virus Disease [9] and was found to be much more complex than what is often assumed in compartment models. Two different consequences may result from this complex coupling: First, the time evolution of the two variables may be completely different and may be characterised by very different levels of predictabilty; Second, our ability to obtain performent models from two variables of the same epidemiological system can also be affected very differently, due to a problem of observability and controllability [41, 45, 46]. This may have direct consequences on the model quality and on its forecasting performances.

Chaos includes extremely diverse catergories of dynamics. Beyond this general property of chaotic feature detected here in many situations, specific chaotic dynamics were found. In particular, a model presenting bistability was obtained for one country (Ghana). This result was completely unexpected, first because bistability is quite rare in non symmetric dynamical systems [47] and no bistable models was ever obtained from observational time series, in particular under environmental conditions. Second, for its epidemiological implications: bistability proves that, under strictly identical conditions (exactly the same equations and parameter values, that is, under exactly the same sanitary, policy, demographic, etc. conditions), the epidemic can evolve on distinct attractors, depending on the attraction basin from which the initial conditions started from, at the very beginning of the outbreak. Therefore, for strictly identical sanitary conditions, two equivalent countries (or even the same country) may experience very different epidemiological situations as a consequence of the early evolution of the epidemic which will lead to one attraction basin or another before converging to its corresponding attractor. Typically, a rapid growth of the number of infected cases at the beginning of the epidemic may directly lead to the attractor corresponding to a situation of quick diffusion of the disease, whereas a careful control of the growth since the earlier beginning of the epidemic (typically by avoiding massive entrance of new cases), may enable to contain the successive outbreaks at a low level.

### Impact of the mitigation strategy

The overall behaviour of the contact number, characterised by a quick decrease at the beginning of the epidemic and followed by complex oscillations, is found to be in rather good agreement with the three indices considered in the study. Considering the details of the variations, the agreement with the reversed Oxford index 100 − *i*_*ox*_ remains convincing only for few countries, all presenting relatively higher health care capacities. It is relevant for South Africa, Tunisia and Kenya, and, although less obviously, for Morocco, Algeria and Egypt. This correspondance is however far to be perfect and numerous reasons can contribute to explain the observed differences.

One important effect that must be pointed out is the inertial effect. It is explained by the time required for social reorganisation after new constraining measures are applied but also after the constraints are relaxed. Communication about the disease in the media (to which the Oxford index accounts for) can also have such an inertial effect. Such a behaviour is clearly illustrated by South Africa where the changes in the stringency index are associated with reversing slope of the contact number rather than abrupt changes.

Several other reasons can be put forward. Contact number involves factors (both objective and subjective) that are not taken into account in the stringency index. Control measures can generate increasing socio-economical incompatibilities with the everyday life making the respect of the measures difficult or sometimes impossible, generating mistrust, and thus fostering behaviours that may facilitate the propagation of the disease. Moreover, the repetitive application of control measures on a long duration will also lead to fatigue and weariness.

Regardless of the impact of the control measures, the new variants that have emerged since the beginning of the pandemic must have influenced the level of the contact number, but also the perception of the disease. Indeed, the emerging variants have shown an increasing capacity of spreading, which may have contributed to increase the contact number. At the mean time, the level of severity and the rate of mortality have also progressively decreased with the successive variants (considerably after omicron variant), reducing the fear of being contaminated. This must have contributed to increase interpeople contacts. Though, increasing trends of the contact number are observed only in very few countries (e.g. Morocco, Tunisia, Lybia). Elsewhere, this influence is not detectable as a general trend but it has probably played a role in the observed oscillations. Indeed, each outbreak event, often associated with new variants, has generated a quick increase of the number of cases and deaths, followed by a decrease in reaction to both the fear deriving from the perception of this increase (media communication effect, or contaminated relatives) and as a result of the control measures specifically put in place. Contrarily to the previous ones, the effect of the Omicron variant was found obvious for most of the countries.

Concerning the specific behaviour observed in Morocco, one main factor can be pointed out. In comparison to the other African countries, Morocco has been far advanced in the vaccination process. In October 2021, 60% of the population had already received at least one dose whereas quite few countries had already passed 20% (Tunisia 42.8%, South Africa, 21.0%, Zimbabwe 20.5% and Lybia 20.1%), and most of them were still under 10% and possibly much lower. The trend observed here is probably associated with the vaccination (70.2% Sputnik / 29.8% Pfizer/Biomoderna in Morocco) which efficacy is probably over-estimated here. This trend cannot be explained by the release of the control measures since no significant variations of the stringency index is observed during this period.

Methodology must also be mentioned to explain the differences. Although quantitative in its formulation, the Oxford index remains mostly qualitative in practice, and cannot be expected to give direct information about the contact number to which it is here compared. Indeed, it is an aggregated index that relates on multiple sub-contributions. Each contributing factor, as well as the weight given to each contributor, cannot be expected to relate simply to the contact number. To this end, its construction is in a large part arbitrary. Moreover, this index is largely based on governmental declarations, but it does neither account for the practical acceptance of the decisions, nor for their follow-up, even less for their unexpected reverse consequences (due to the misacceptance of the constraining measures, mistrust of the vaccines, etc.). In other words, despite its genuine interest, this index must be considered with caution for the present comparison.

On the long term, most of the control measures have had a real impact on people life. Indeed, it is observed that the progressively decreasing level of stringency (positive trend of the reverse Oxford index) is in agreement with the trends of the two mobility indices, and for all the countries. Though, this effect is not obvious in the contact number. In other words, mobility has apparently been a minor contributor to the propagation of the disease.

Of course, our estimates of the contact number will also present limitations since based on observational data (the epidemiologic time series), which, themselves, can suffer from various observational limitations and biases, and because their reconstruction also relies on underlying hypotheses. However, the effect of underreporting is in principle largely attenuated (correction applied based on the health system capacity), and the error resulting from poorly known parameters is found rather moderate in amplitude (≤ ±0.03 contact per person and per day).

These limitations in the comparison being kept in mind, the efficacy of the control measures appears rather contrasted. On one hand, the quick decrease of the contact number at the beginning of the pandemic highlights the overall efficacy of the control measures, and the following oscillations reveal that some of them have contributed to limit temporarily and efficiently the development of the successive outbreaks. On the other hand, the progressive release of the original high level of stringency has not revealed any effect on the long term. The usefulness of maintaining a high level of control measures on long duration is therefore questioned. However, this observation seems consistent with the situation of bistability unveiled by one of the models: Although possibly too much stringent, the control measures have at least enabled to maintain the epidemic on a dynamics of relatively low level of propagation.

## Supporting information

S1 Fig Geographical distribution of COVID-19 with weighting *w*_*A*_ and *w*_*B*_ applied separately. Geographical distribution of the numbers of COVID-19 cases (A) and deaths (B) per thousand inhabitants with health system correction applied based on the number of hospital beds per inhabitant; And of the numbers of COVID-19 cases (C) and deaths (D) per thousand inhabitants of 60 years old and over. Map created using Plotly and Mapbox. https://plotly.com/python/map-configuration.
(TIFF)

S2 Fig Time series of cases. Observed and pre-processed time series of COVID-19 cases per million inhabitants from January 1st 2020 (day 1) for seventeen African countries. The thin lines correspond to the original observations uncorrected (in blue) or corrected (in black) from the health system bias. Pre-processed time series are provided in thick lines with the one sigma error bar associated with it (in red).
(TIFF)

S3 Fig Time series of deaths. Observed and pre-processed time series of COVID-19 deaths per million inhabitants from January 1st 2020 (day 1) for seventeen African countries. The thin lines correspond to the original observations uncorrected (in blue) or corrected (in black) from the health system bias. Pre-processed time series are provided in thick lines with the one sigma error bar associated with it (in red).
(TIFF)

S4 Fig Phase portraits for the dynamics of cases. Original (in black) and modelled (in green) differential phase portraits, in (*I*_1_, *I*_2_) projection, for the dynamics of COVID-19 cases, for 6 African countries. Original phase portrait is reconstructed for the period 22 January 2020 to 21 June 2021 where solid lines were used for modelling, dashed lines for validation.
(JPEG)

S5 Fig Phase portraits for the dynamics of deaths. Original (in black) and modelled (in green) differential phase portraits, in (*D*_1_, *D*_2_) projection, for the dynamics of COVID-19 deaths, for 8 African countries. Original phase portrait is reconstructed for the period 22 January 2020 to 21 June 2021 where solid lines were used for modelling, dashed lines for validation.
(JPEG)

S6 Fig Absolute forecasting error growth |*e*(*τ*)| of the daily new cases of COVID-19, as a function of the prediction time *τ*, for models obtained for twelve countries. For each country, the error growth is reported for 250 initial conditions equally distributed over the modelling period, on a prediction horizon of 0 to 20 days (green lines). Three confidence levels (in black) are provided corresponding to the percentiles 75% (dashed lines), 90% (dashed-dotted line) and 95% (dotted line). The same confidence levels are provided for the validation window (in orange).
(TIFF)

S7 Fig Absolute forecasting error growth |*e*(*τ*)| of the daily COVID-19 deaths, as a function of the prediction time *τ*, for models obtained for nine countries. See S6 Fig for details.
(TIFF)

S8 Fig First return maps of models for cases. Maps were reconstructed for (A) Egypt cases model with *κ* = 1. (in black), *κ* = 1.25 (in magenta) and *κ* = 1.7 (in green); (B) Ethiopia cases model with *κ* = 0.91 (in green) and *κ* = 0.93 (in blue); (C) Ghana-1 cases model (Eqs. 33) with *κ* = 0.85 but using different initial conditions (magenta and green), and Ghana-2 model (Eqs. 41) (in orange); (D) Kenya cases model with *κ*_1_ = *κ*_2_ = 1. (in green), (*κ*_1_, *κ*_2_) = (1.08, 0.9901) (in red) and (*κ*_1_, *κ*_2_) = (1, 0.9882353) (in blue); (E) Namibia cases model with *κ*_1_ = *κ*_2_ = *κ*_3_ = 1. (in black), (*κ*_1_, *κ*_2_, *κ*_3_ = (1.06, 1., 1.) (in green) and (*κ*_1_, *κ*_2_, *κ*_3_ = (1., 0.9, 1.12) (in red); and (F) South Africa cases model (black only). Corresponding equations and initial conditions are provided in S3 Appendix (Section 1).
(JPEG)

S9 Fig First return maps of models for deaths. Maps were reconstructed for (A) Algeria deaths model with *κ*_1_ = *κ*_2_ = 1. (in black) and *κ*_1_ = 1. and *κ*_2_ = 1.2 (in green); (B) Cameroon deaths model; (C) Egypt deaths model; (D) Namibia deaths model; and (E) Zimbabwe deaths model. Corresponding equations and initial conditions are provided in S3 Appendix (Section 2).
(JPEG)

S10 Fig Differential phase portraits in (*I*_1_, *I*_2_) projection of the daily new cases dynamics. Observations (black lines) and models (green lines).
(JPEG)

S11 Fig Differential phase portraits in (*D*_1_, *D*_2_) projection of the deaths dynamics. Observations (black lines) and models (green lines).
(JPEG)

S1 File Database. This file provides the total number of cases and deaths, on 10 January 2022, for 150 countries in the world, as provided by the *Center for System Science and Engineering* of the *John Hopkins University* [32]. It also includes the number of hospital beds per inhabitants and the number of people of 60-year old and older from the *United Nations* [30] and the *World Bank* [31], respectively.
(XLS)

S1 Appendix Estimating the contact number. The number of contact per person and per day is an important parameter because this number plays a key role in the transmission of a virus during an epidemic. Here, a reformulation of the equations of an epidemic is used to reconstruct *β*(*t*) the number of contact per person and per day from *I*(*t*) the number of new cases per day and *V*(*t*) the number of vaccination at time *t*. All the details of these reformulation are provided in the present Appendix. To test its validy, the approach is applied to the dynamics of a 7-compartment model in S2 Appendix in order to show that, although based on a simple formulation, this formulation can apply to dynamics of higher complexity.
(PDF)

S2 Appendix Two applicative scenarios. A seven-compartment model involving *S* (susceptible), *E* (exposed), *i*^*S*^ and *i*^*A*^ (symptomatic and asymptomatic cases), *R* (recovered), *V* (vaccinated) and *D* dead comparments was used to simulate the epidemic with the number of contact varying as a function of time under two different scenarios. Based on the number of new cases per day *I*(*t*) and the number of vaccination *V*(*t*) (see S1 Appendix for details), it was proven possible to reconstruct the time evolution of the number of contact per day and per person for the two scenarios.
(PDF)

S3 Appendix Models equations. All the global models obtained in the present study are based on the same general algebraic structure given in Eq 5 with *n* = 3. The detailed definition of the polynomial *Q*(*X*_1_, *X*_2_, *X*_3_) provided in the present Appendix are thus sufficient to have the full model description. Note that the initial conditions are also provided.
(PDF)

S1 Table Dynamical regimes of the models. The models dynamics was investigated considering 100 000 integration time steps of 0.1 day each (corresponding to a duration of 24 years). Metastable is mentioned when the integration could be checked on 20 000 time steps (∼6 years) only. P1, P2 and P5 refer to period cycles of period one, two and five, respectively. Toroidal chaos refers to chaotic attractors structured around, and bounded by, a toroidal structure (see [48] for details).
(PDF)

S2 Table Models forecasting performances. Models forecasting performances: forecasting error (in % of the maximum value observed during the modelling period), for a 10 day prediction horizon (90% confidence level) of daily cases and deaths per million inhabitants. Error level is provided for the validation window and also (in brackets) for the modelling window. The modelling and validation (in brackets) period length (in day) are also provided.
(PDF)
