# Supplementary figures and images for: COVID-19 in Africa: Underreporting, demographic effect, chaotic dynamics, and mitigation strategy impact
Source: PLoS Negl Trop Dis. 2022 Sep 16;16(9):e0010735. doi: 10.1371/journal.pntd.0010735 (PMC9518880; doi:10.1371/journal.pntd.0010735)

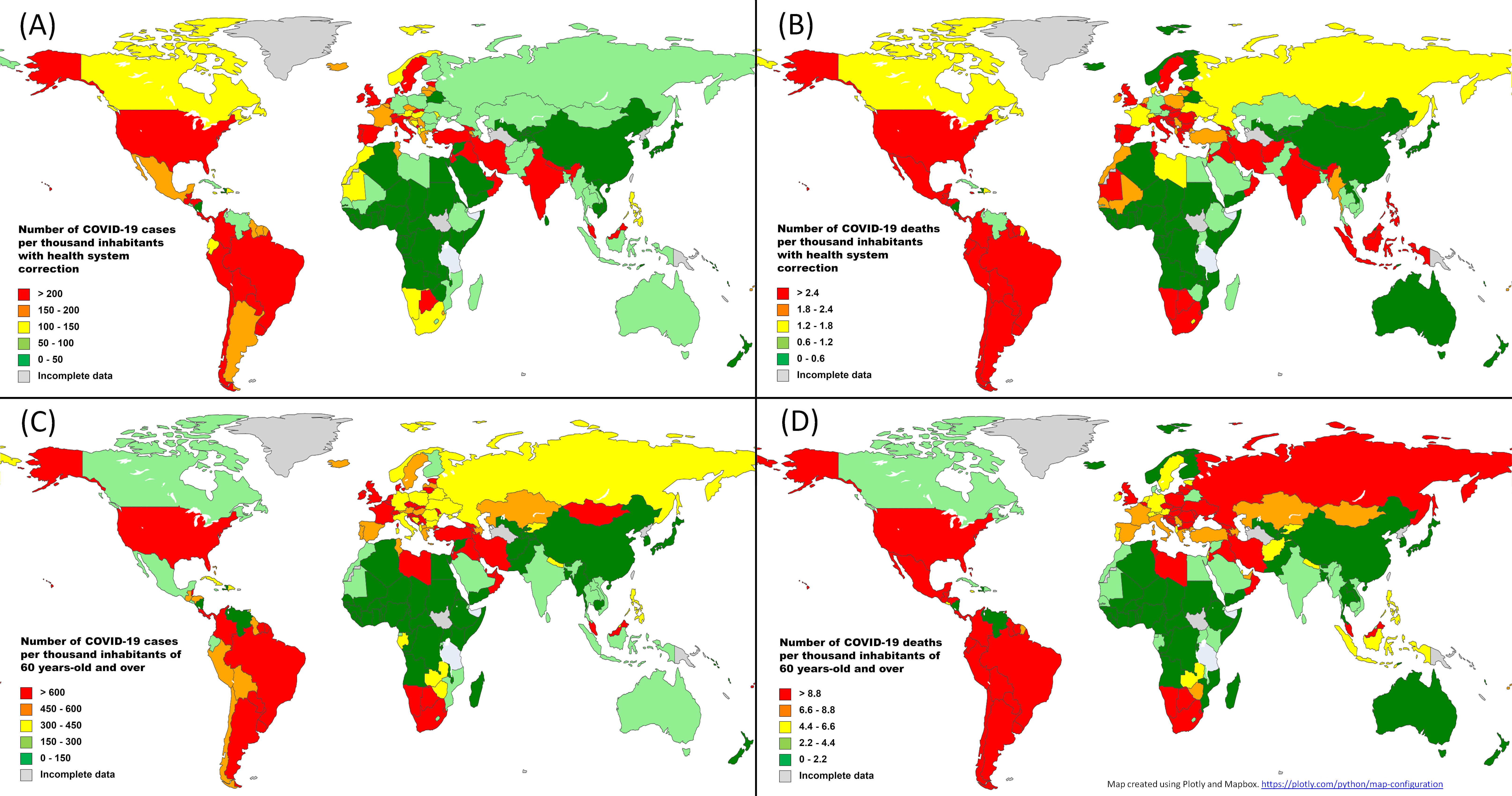

Supplement: S1 Fig — Geographical distribution of the numbers of COVID-19 cases (A) and deaths (B) per thousand inhabitants with health system correction applied based on the number of hospital beds per inhabitant; And of the numbers of COVID-19 cases (C) and deaths (D) per thousand inhabitants of 60 years old and over. Map created using Plotly and Mapbox. https://plotly.com/python/map-configuration. (TIFF) [file pntd.0010735.s001.tiff]

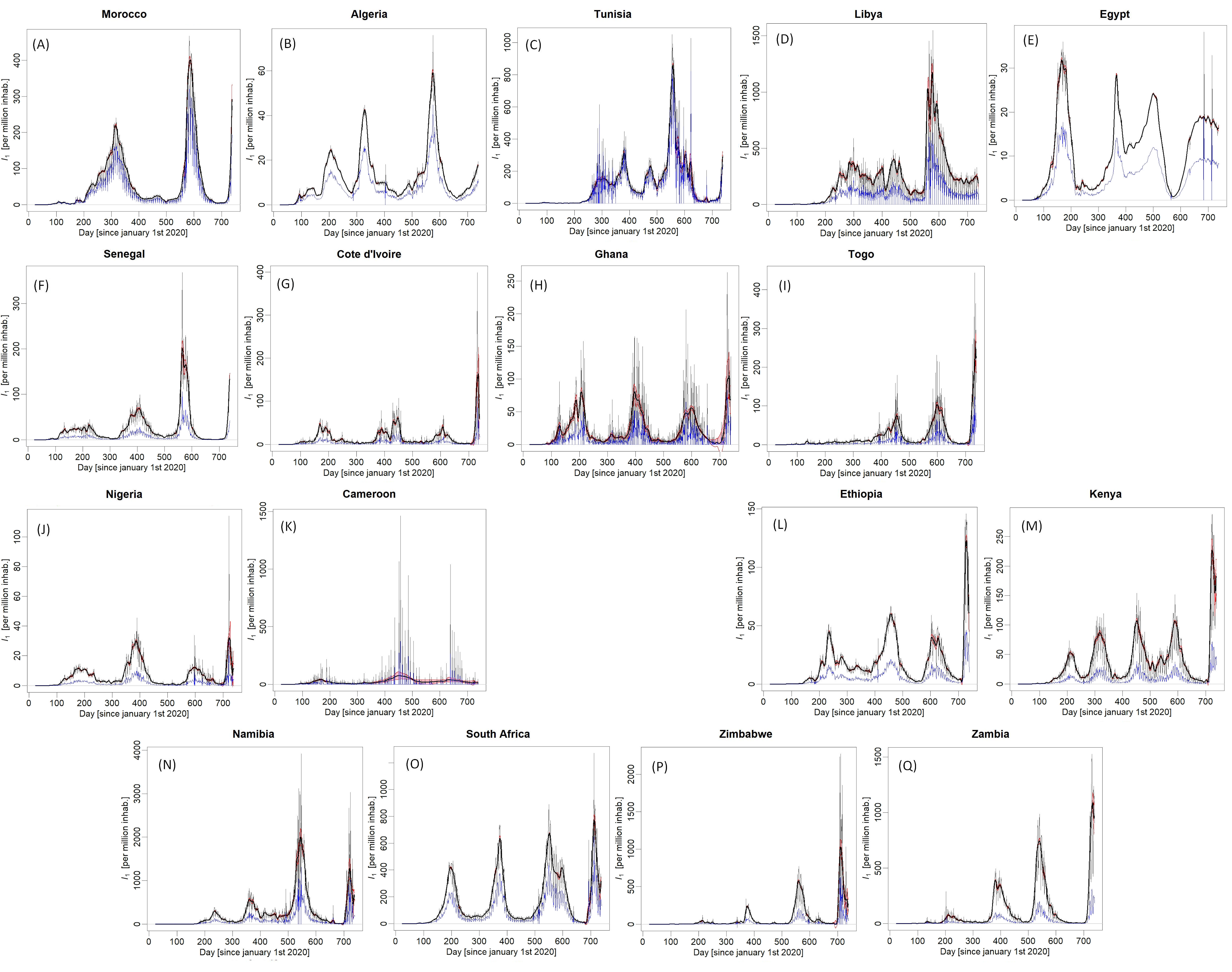

Supplement: S2 Fig — Observed and pre-processed time series of COVID-19 cases per million inhabitants from January 1st 2020 (day 1) for seventeen African countries. The thin lines correspond to the original observations uncorrected (in blue) or corrected (in black) from the health system bias. Pre-processed time series are provided in thick lines with the one sigma error bar associated with it (in red). (TIFF) [file pntd.0010735.s002.tiff]

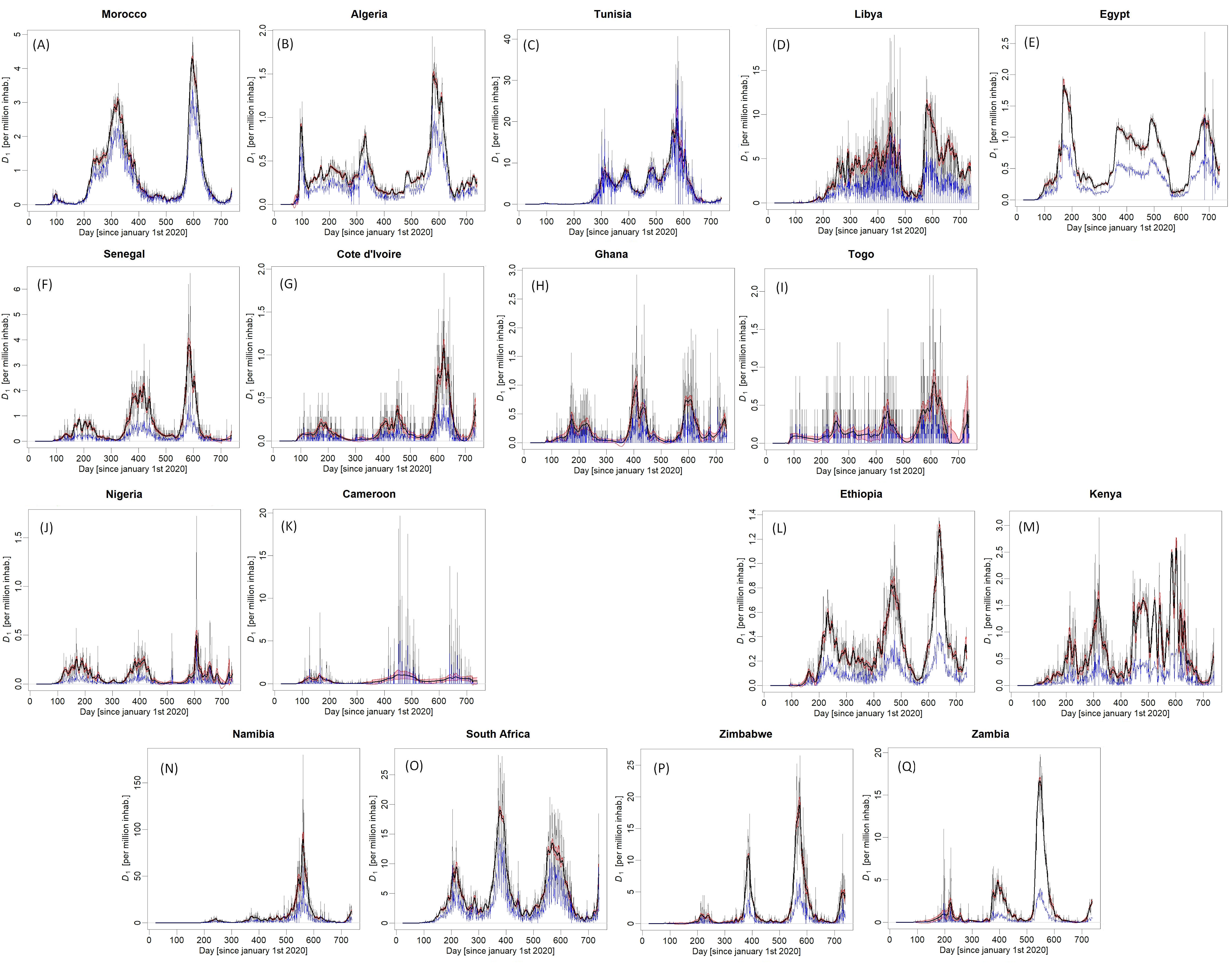

Supplement: S3 Fig — Observed and pre-processed time series of COVID-19 deaths per million inhabitants from January 1st 2020 (day 1) for seventeen African countries. The thin lines correspond to the original observations uncorrected (in blue) or corrected (in black) from the health system bias. Pre-processed time series are provided in thick lines with the one sigma error bar associated with it (in red). (TIFF) [file pntd.0010735.s003.tiff]

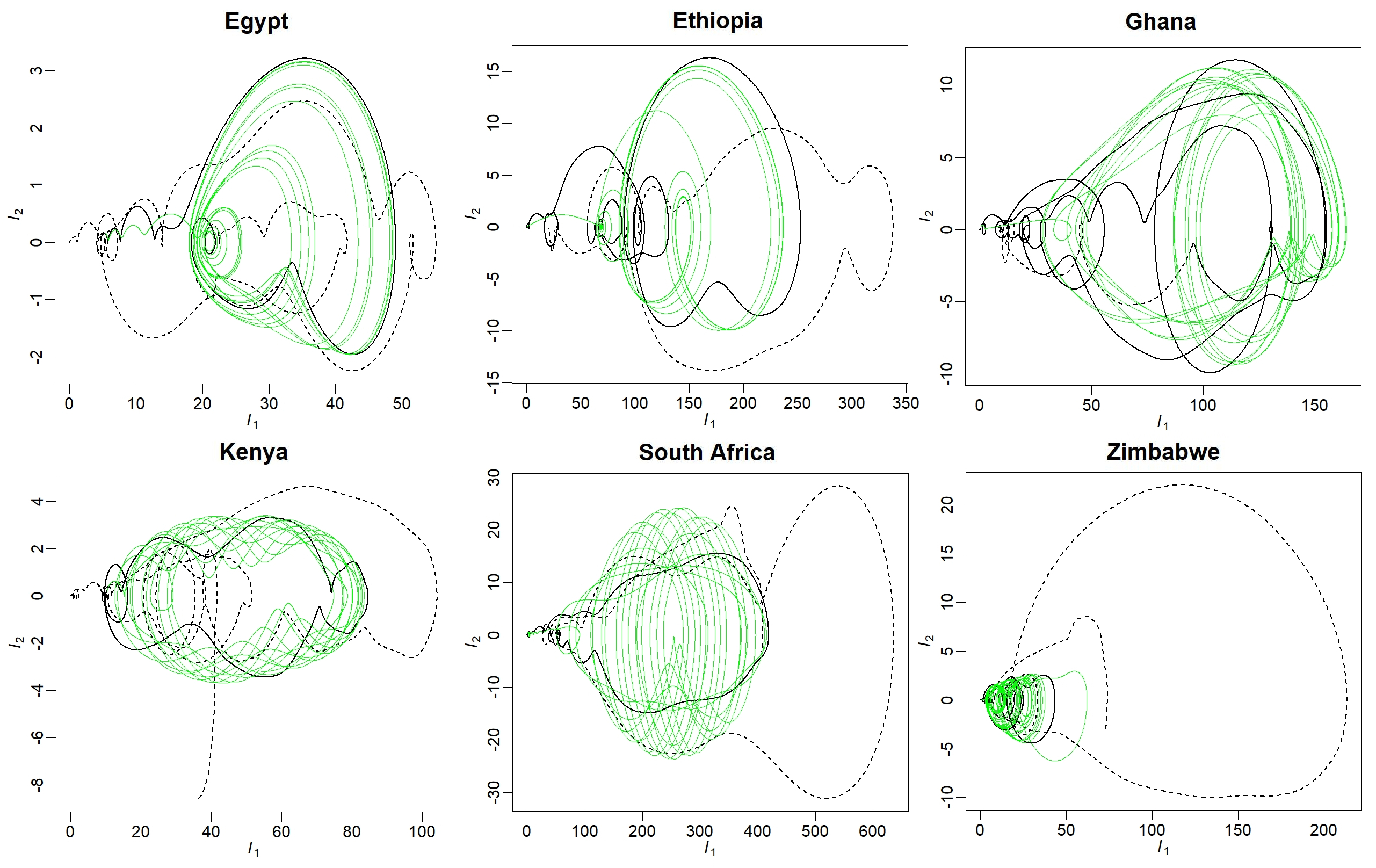

Supplement: S4 Fig — Original (in black) and modelled (in green) differential phase portraits, in (I1, I2) projection, for the dynamics of COVID-19 cases, for 6 African countries. Original phase portrait is reconstructed for the period 22 January 2020 to 21 June 2021 where solid lines were used for modelling, dashed lines for validation. (JPEG) [file pntd.0010735.s004.jpeg]

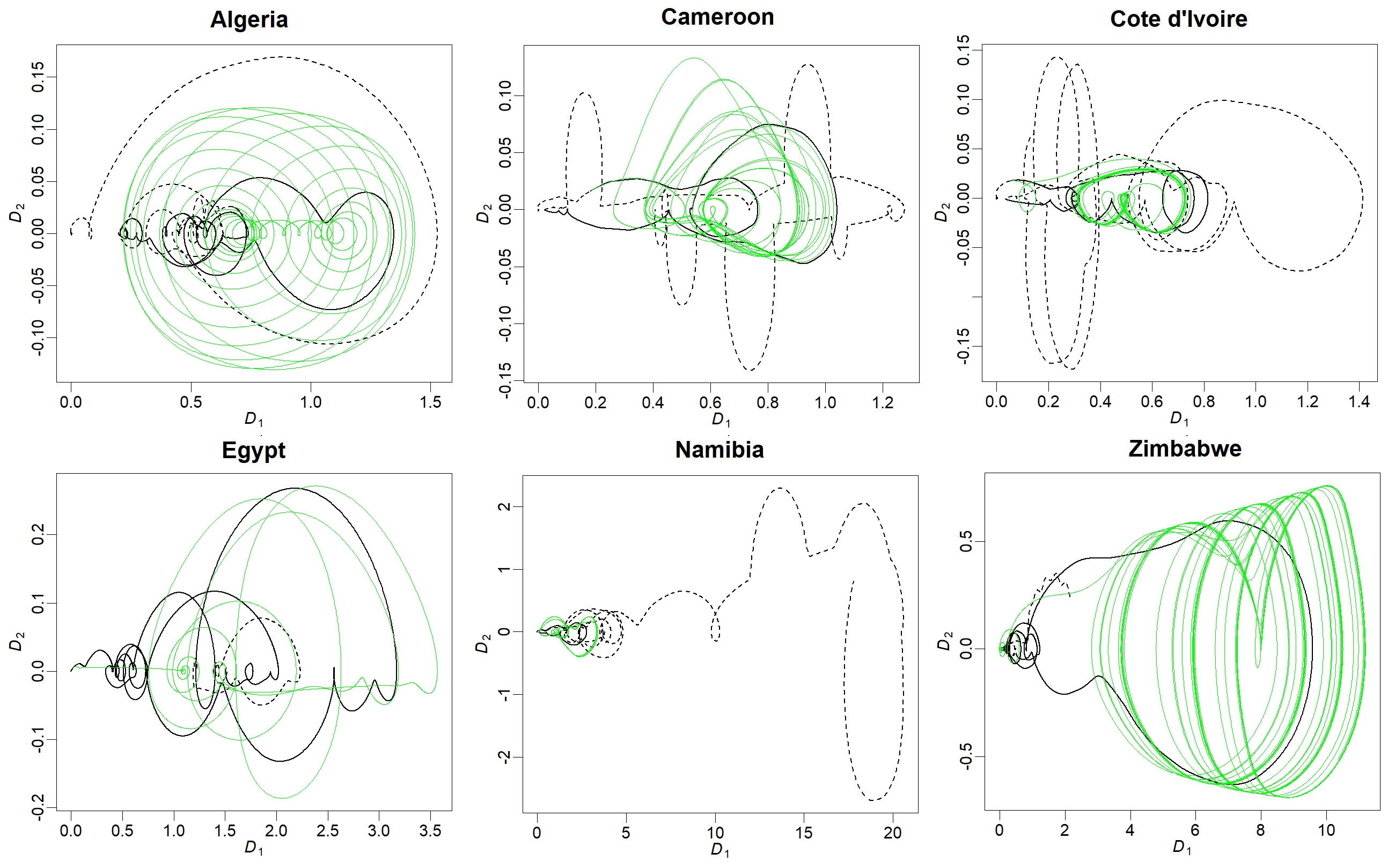

Supplement: S5 Fig — Original (in black) and modelled (in green) differential phase portraits, in (D1, D2) projection, for the dynamics of COVID-19 deaths, for 8 African countries. Original phase portrait is reconstructed for the period 22 January 2020 to 21 June 2021 where solid lines were used for modelling, dashed lines for validation. (JPEG) [file pntd.0010735.s005.jpeg]

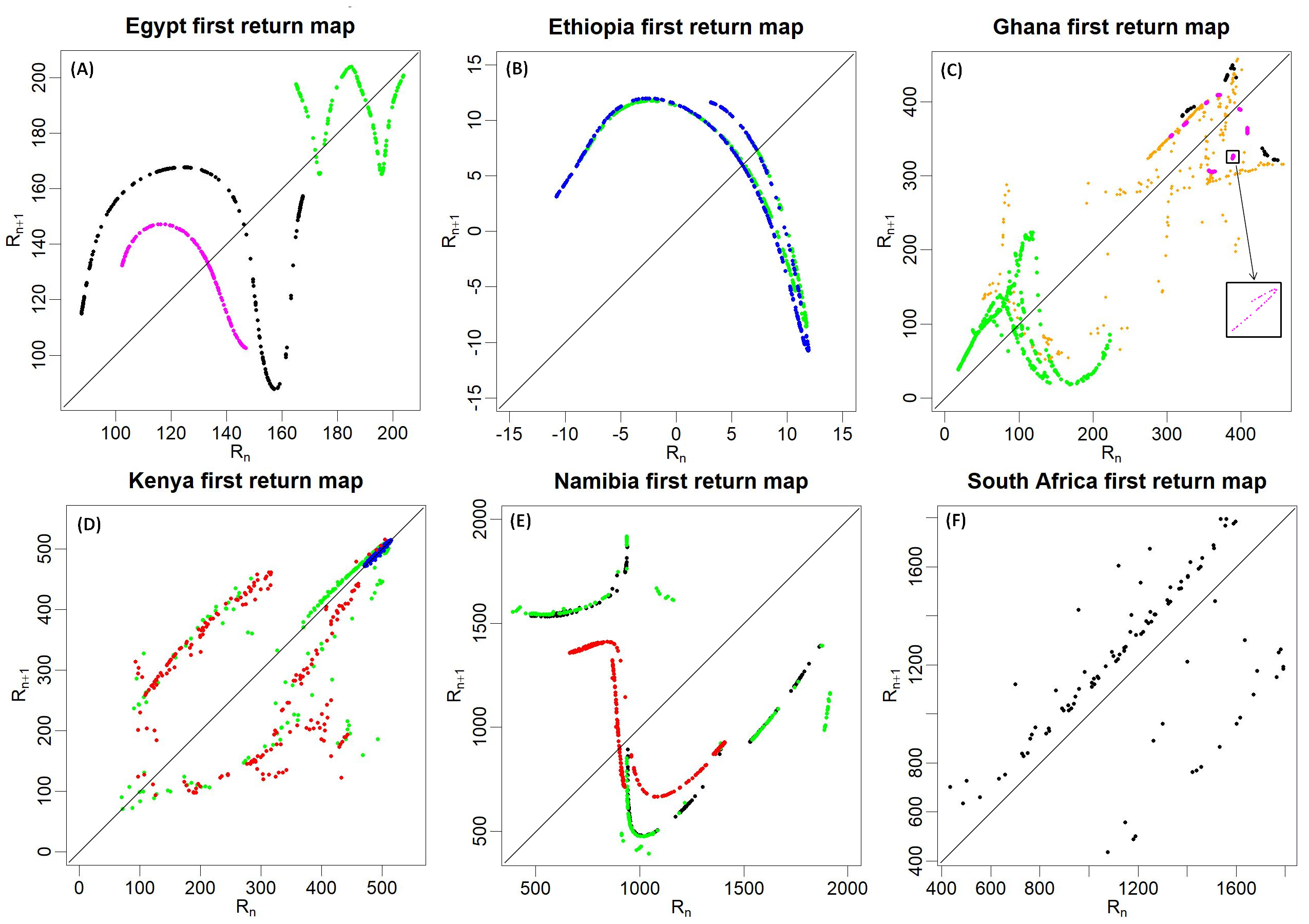

Supplement: S8 Fig — Maps were reconstructed for (A) Egypt cases model with κ = 1. (in black), κ = 1.25 (in magenta) and κ = 1.7 (in green); (B) Ethiopia cases model with κ = 0.91 (in green) and κ = 0.93 (in blue); (C) Ghana-1 cases model (Eqs. 33) with κ = 0.85 but using different initial conditions (magenta and green), and Ghana-2 model (Eqs. 41) (in orange); (D) Kenya cases model with κ1 = κ2 = 1. (in green), (κ1, κ2) = (1.08, 0.9901) (in red) and (κ1, κ2) = (1, 0.9882353) (in blue); (E) Namibia cases model with κ1 = κ2 = κ3 = 1. (in black), (κ1, κ2, κ3 = (1.06, 1., 1.) (in green) and (κ1, κ2, κ3 = (1., 0.9, 1.12) (in red); and (F) South Africa cases model (black only). Corresponding equations and initial conditions are provided in S3 Appendix (Section 1). (JPEG) [file pntd.0010735.s008.jpeg]

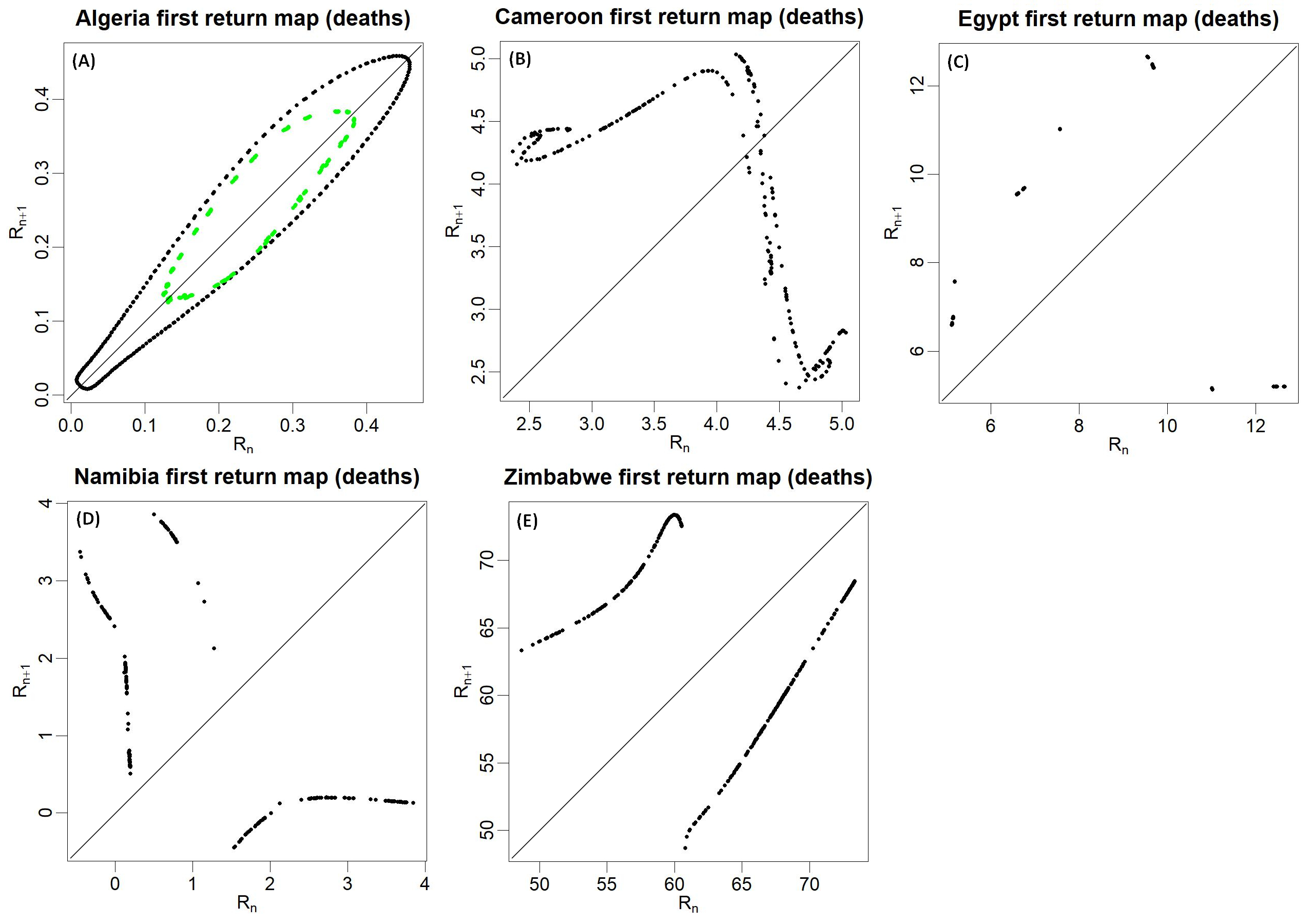

Supplement: S9 Fig — Maps were reconstructed for (A) Algeria deaths model with κ1 = κ2 = 1. (in black) and κ1 = 1. and κ2 = 1.2 (in green); (B) Cameroon deaths model; (C) Egypt deaths model; (D) Namibia deaths model; and (E) Zimbabwe deaths model. Corresponding equations and initial conditions are provided in S3 Appendix (Section 2). (JPEG) [file pntd.0010735.s009.jpeg]

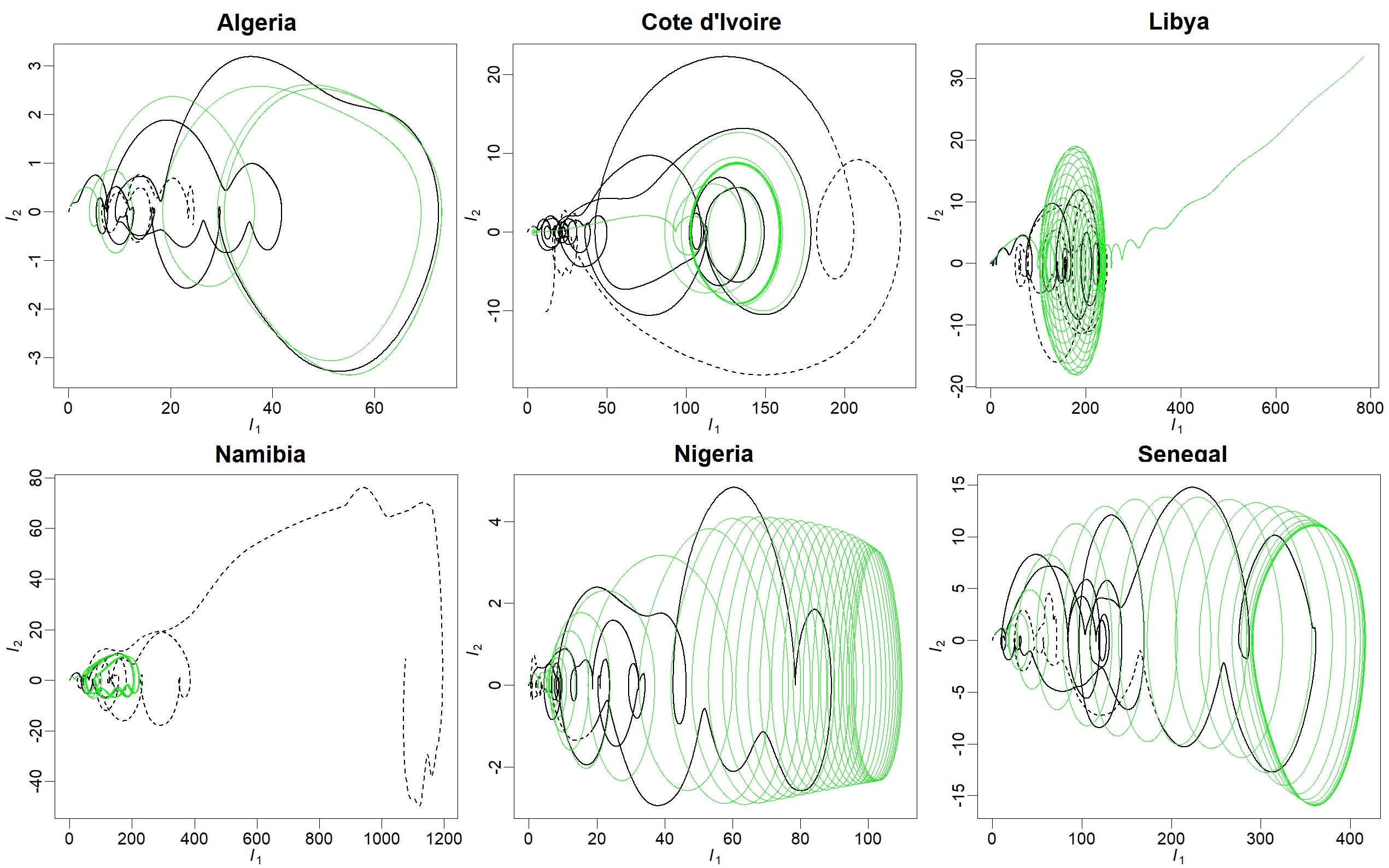

Supplement: S10 Fig — Observations (black lines) and models (green lines). (JPEG) [file pntd.0010735.s010.jpeg]

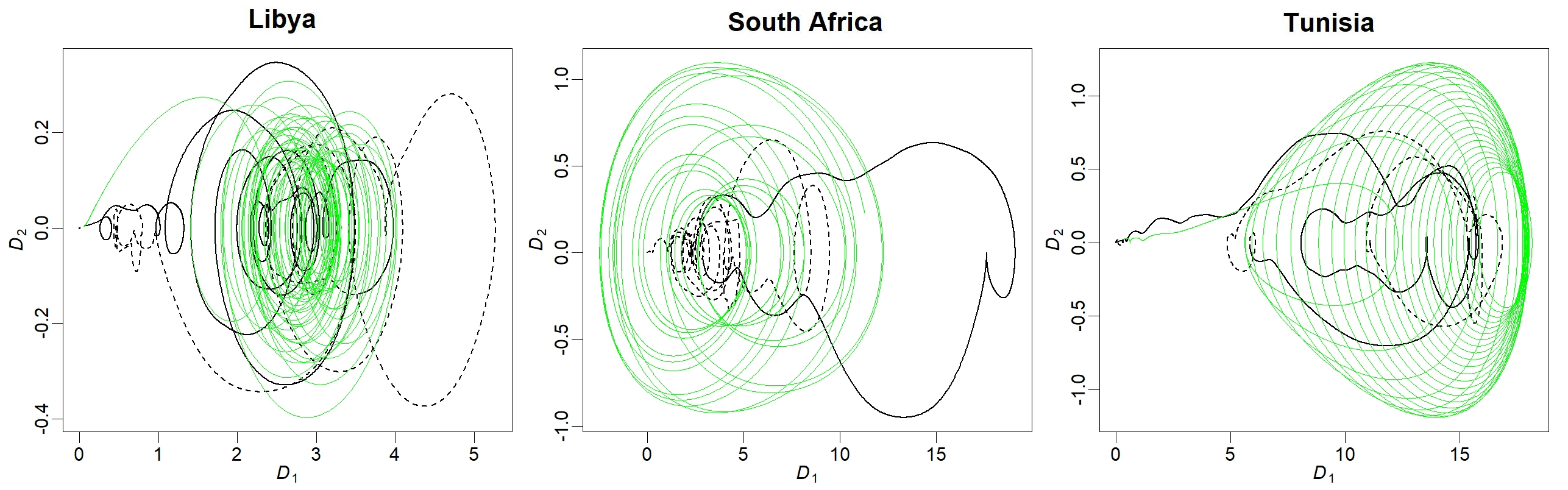

Supplement: S11 Fig — Observations (black lines) and models (green lines). (JPEG) [file pntd.0010735.s011.jpeg]
