# Supplementary material for: COVID-19 in Africa: Underreporting, demographic effect, chaotic dynamics, and mitigation strategy impact
Source: PLoS Negl Trop Dis. 2022 Sep 16;16(9):e0010735. doi: 10.1371/journal.pntd.0010735 (PMC9518880; doi:10.1371/journal.pntd.0010735)
Supplement: S1 Appendix — The number of contact per person and per day is an important parameter because this number plays a key role in the transmission of a virus during an epidemic. Here, a reformulation of the equations of an epidemic is used to reconstruct β(t) the number of contact per person and per day from I(t) the number of new cases per day and V(t) the number of vaccination at time t. All the details of these reformulation are provided in the present Appendix. To test its validy, the approach is applied to the dynamics of a 7-compartment model in S2 Appendix in order to show that, although based on a simple formulation, this formulation can apply to dynamics of higher complexity. (PDF) [file pntd.0010735.s013.pdf]

S1 Appendix for

Covid-19 in Africa: underreporting, demographic effect, chaotic dynamics, and mitigation strategies impact

Authors: Nathan Thenon[1,2†], Marisa Peyre[2], Mireille Huc[1], François Roger[2], Sylvain Mangiarotti[1*†].

[*]To whom correspondence should be sent: sylvain.mangiarotti@ird.fr

†These authors contributed equally to this work

Appendix S1 - Estimating the contact number

A. Averaged number of contact estimated from the new cases

The governing equations of the observed dynamics are generally unknown. Here, we use a simplified formulation of the epidemic to explain how to reconstruct the efficacy of the non pharmaceutical measures from a single variable commonly observed under real conditions: the number of new cases per day $I(t)$. It will be later applied to the dynamics of the SEi^{2}RD model (see S2 Appendix) in order to investigate if, although based on a simple formulation, this formulation can apply to a dynamics of higher complexity.

The SEiRD model is commonly used in epidemiology where S stands for Susceptible, E for Exposed and i for infected, R for recovered and D for dead people at time t:

$$\begin{cases} \frac{dS_t}{dt} = -\frac{\beta}{N_t} S_t i_t \\ \frac{dE_t}{dt} = +\frac{\beta}{N_t} S_t i_t - \alpha E_t \\ \frac{di_t}{dt} = +\alpha S_t i_t - \upsilon i_t - m i_t \\ \frac{dR_t}{dt} = +\upsilon i_t \\ \frac{dD_t}{dt} = +m i_t , \end{cases} \tag{1}$$

and $N_t = S_t + E_t + i_t + R_t$ the total population at time t. Considering that D is only a by product of the SEIR model and that the variations of N are very small (and also slow) in comparison to the other variations, this dynamics can be reduced to the three equations

$$\begin{cases} \frac{dS_t}{dt} = -\frac{\beta}{N_t} S_t i_t \\ \frac{dE_t}{dt} = +\frac{\beta}{N_t} S_t i_t - \alpha E_t \\ \frac{di_t}{dt} = +\alpha S_t i_t - \upsilon i_t - m i_t , \end{cases} \tag{2}$$

and then, assuming that the exposure plays as a simple delay between the infection stage and infected stage, the system can be rewritten in two equations

$$\begin{cases} \frac{dS_t}{dt} = -\frac{\beta}{N} S_t i_t \\ \frac{di_t}{dt} = +\frac{\beta}{N} S_{t-\tau} i_{t-\tau} - \upsilon i_t - m i_t , \end{cases} \tag{3}$$

with τ the average time delay before an exposed people becomes infected (be he symptomatic or not).

The number of daily new cases $I_t^{(1)}$ (where the number in bracket denotes the first derivative) is thus given by

$$I_t^{(1)} = +\frac{\beta}{N} S_{t-\tau} i_{t-\tau} \,, \tag{4}$$

and its cumulative number by

$$I_t^{(0)} = \int +\frac{\beta}{N} S_{t-\tau} i_{t-\tau} dt \,. \tag{5}$$

Rewriting the first equation of Eqs. (3) into

$$S_{t-\tau} = -\frac{N}{\beta i_{t-\tau}} \frac{dS_{t-\tau}}{dt} \,,$$

we get

$$S_{t-\tau} = S_{t=0} - I_t^{(0)} \,, \tag{6}$$

by integrating Eq. (5). Replacing Eq. (6) in Eq. (4) we get

$$I_t^1 = +\frac{\beta}{N} \left(S_{t=0} - I_t^{(0)} \right) i_{t-\tau} \,. \tag{7}$$

The exposure ratio can thus be written

$$\beta = \frac{N \, I_t^{(1)}}{\left(S_{t=0} - I_t^{(0)} \right) i_{t-\tau}} \,. \tag{8}$$

This formula remains valid locally if β is varying with time. As i_t is not always available, it can be estimated from $I_t^{(1)}$ such as

$$\hat{i}_t = \int_{t-T}^{t} I_t^{(1)} dt \,, \tag{9}$$

with T the characteristic time of the disease, so that $\hat{\beta}(t)$ can be entirely deduced from the single variable $I_t^{(1)}$. Taking into account the number of vaccination V_t at time *t* (if available), we get

$$\hat{\beta}(t) = \frac{N \, I_t^{(1)}}{\left(S_{t=0} - I_t^{(0)} - V_t \right) i_{t-\tau}} \,, \tag{10}$$

with $i_{t-\tau} \neq 0$. Note that i_t is composed of both symptomatic and asymptomatic compartment here ($i_t = i_t^S + i_t^A$) and may thus be difficult to estimate practically since asymptomatic people are by definition difficult to count. However, this limitation will only have a marginal effect on the estimate of $\hat{\beta}(t)$ because it will act similarly onto $I_t^{(1)}$. To avoid singularities, $\hat{\beta}(t)$ is estimated only if $i_{t-\tau} \geq 100$. This formulation was tested on synthetic cases and revealed a systematic overestimation (in the range 1.28 to 1.42) when varying the infected period T_i from 5 to 7.5 days (see Table A in the present S1 Appendix). A correction coefficient $\xi = 0.75$ such as

$$\hat{\beta}_t = \xi \beta_t \tag{11}$$

was thus applied to get more realistic estimates of the average number of contact per person per time.

Table A:

T_{i}	4.5	4.75	5	5.5	6	6.5	7	7.25	7.5
τ	3.2	3.4	3.6	3.9	4.3	4.6	5.0	5.2	5.4
ξ	0.63	0.67	0.70	0.73	0.78	0.78	0.78	0.75	0.70
$1/\xi$	1.59	1.50	1.42	1.37	1.28	1.28	1.28	1.33	1.42

B. Recovery and Mortality rates

The recovery rate υ and the mortality rate m can also be deduced from i_t (deduced from $I_t^{(1)}$ using Eq. 9) and $R_t^{(1)}$ (to be estimated numerically from R_t)

$$\hat{\upsilon}(t) = \frac{R_t^{(1)}}{i_t}, \tag{12}$$

or $D_t^{(1)}$ (to be estimated numerically from D_t)

$$\hat{m}(t) = \frac{D_t^{(1)}}{i_t}, \tag{13}$$

Here also, the contribution of the asymptomatic cases are compensated.

C. Effective reproduction number

Since the original dynamics is a priori unknown, three formulations of the basic reproduction number $\mathbf{R}_0$ were investigated, based on SIR model

$$\mathbf{R}_0^{\mathrm{SIR}} = \frac{\beta}{\upsilon}, \quad (14)$$

SIRD model

$$\mathbf{R}_0^{\mathrm{SIRD}} = \frac{\beta}{\upsilon + m}, \quad (15)$$

and SEi2RD model hypotheses:

$$\mathbf{R}_0^{\mathrm{SEi2RD}} = \frac{\beta}{\upsilon + m} + \frac{(1-p)m}{\upsilon + m}. \quad (16)$$

The three formulations were tested to estimate the effective reproduction number

$$\mathbf{R}_t = \mathbf{R}_0 \times \frac{\left(S_{t=0} - I_t^{(0)} - V_t\right)}{N_t}, \quad (17)$$

the SIRD formulation was found the more realistic when applied to the synthetic data. The following formulation

$$\hat{\mathbf{R}}_t = \frac{\beta_t}{\upsilon_t + m_t} \times \frac{\left(S_{t=0} - I_t^{(0)} - V_t\right)}{N_t}, \quad (18)$$

was thus preferred – in practice – to estimate the effective reproduction number. Other formulations were kept to estimate the error resulting from this lack of knowledge.
