# Supplementary material for: COVID-19 in Africa: Underreporting, demographic effect, chaotic dynamics, and mitigation strategy impact
Source: PLoS Negl Trop Dis. 2022 Sep 16;16(9):e0010735. doi: 10.1371/journal.pntd.0010735 (PMC9518880; doi:10.1371/journal.pntd.0010735)
Supplement: S2 Appendix — A seven-compartment model involving S (susceptible), E (exposed), iS and iA (symptomatic and asymptomatic cases), R (recovered), V (vaccinated) and D dead comparments was used to simulate the epidemic with the number of contact varying as a function of time under two different scenarios. Based on the number of new cases per day I(t) and the number of vaccination V(t) (see S1 Appendix for details), it was proven possible to reconstruct the time evolution of the number of contact per day and per person for the two scenarios. (PDF) [file pntd.0010735.s014.pdf]

S2 Appendix for

Covid-19 in Africa: underreporting, demographic effect, chaotic dynamics, and mitigation strategies impact

Authors: Nathan Thenon[1,2†], Marisa Peyre[2], Mireille Huc[1], François Roger[2], Sylvain Mangiarotti[1*†].

[*]To whom correspondence should be sent: sylvain.mangiarotti@ird.fr

†These authors contributed equally to this work

Appendix S2 - Two applicative scenarios

A compartment model with a weekly exogenous social forcing

Models including an exposed compartment, such as the SEiR models, are commonly used in epidemiology. For the present context, the SEi^{2}RVD formulation

$$\begin{cases} \dfrac{dS_t}{dt} = -\dfrac{\beta_t^S}{N} S_t i_t^S - \dfrac{\beta_t^A}{N} S_t i_t^A - v_t \\ \dfrac{dE_t}{dt} = +\dfrac{\beta_t^S}{N_t} S_t i_t^S + \dfrac{\beta_t^A}{N_t} S_t i_t^A - \left(\alpha^S + \alpha^A\right) E_t^S \\ \dfrac{di_t^S}{dt} = +\alpha^S E_t - \upsilon^S i_t^S - m\, i_t^S \\ \dfrac{di_t^A}{dt} = +\alpha^A E_t - \upsilon^A i_t^A \\ \dfrac{dR_t}{dt} = +\upsilon^S i_t^S + \upsilon^a i_t^A \\ \dfrac{dV_t}{dt} = +v_t \\ \dfrac{dD_t}{dt} = +m\, i_t^S , \end{cases} \qquad (1)$$

with two infected compartments, is used, where S_t stands for the Susceptible, E_t for the Exposed, i_t^S for the symptomatic infected, i_t^A for the asymptomatic infected, R_t for the recovered, V_t for the vaccinated, and D_t for dead people, each at time t, with $N_t = S_t + E_t + i_t + R_t + V_t$ the total population (close to constant since $D_t << N_t$ from the beginning to the end of the epidemic). The average number of contact per person per time β is considered here as a function of time, and can be different for symptomatic and asymptomatic people. It is defined as

$$\beta_t^S = \beta_P^S \varsigma_{DOW}^S , \qquad (2)$$

for symptomatic and

$$\beta_t^A = \beta_P^A \varsigma_{DOW}^A , \qquad (3)$$

for asymptomatic people with β_P^S and β_P^A the values of the exposure ratio for respectively symptomatic and asymptomatic people which can vary depending on the mitigation strategies (see Table A in the present S2 Appendix), and ς_{DOW}^S and ς_{DOW}^A two periodic functions with a fixed value for each Day of Week (see Table B in the present S2 Appendix).

The daily number of new cases is directly given by

$$I_t^{(1)} = \left(\alpha^S + \alpha^A\right) E_t^S , \qquad (4)$$

and the cumulative number of new cases

$$I_t^{(0)} = \int \left(\alpha^S + \alpha^A\right) E_t^S dt , \qquad (5)$$

can be deduced from it.

Two scenarios have been built arbitrarily to test the approach, each including successive differentiated periods characteristic of different mitigation strategies. In both cases, no strategy is applied during the earlier development **A** of the epidemic, the two coefficients β_P^S and β_P^A are then at their maximum. The exposure is assumed to be significantly lower with asymptomatic in comparison to symptomatic ones by a factor 0.75 [1]. A stringent strategy is applied during period **B** to stop the exponential propagation of the disease, leading to lower values. Constrains are partially released during the period **C**, but these are reinforced again in period **D** due to a restart, constrains are released again in period **E**. Non pharmaceutical measures are then slightly reinforced in last period **F** to avoid any restart. In scenario 2, a differentiated strategy is applied leading to differentiated contact numbers for symptomatic and asymptomatic people.

Table A: Reference exposure ratio corresponding to the different mitigation strategies for the six successive periods **A** to **F**.

Period	**A**	**B**	**C**	**D**	**E**	**F**
			Scenario 1			
Window	0-80	80-110	110-200	200-250	250-350	350-500
Length	80	30	90	50	100	150
β_P^S	0.41	0.06	0.23	0.13	0.19	0.41
β_P^A	0.41	0.06	0.23	0.13	0.19	0.41
β_P^A / β_P^S	1.	1.	1.	1.	1.	1.
v_1	0	0	0	0	150	300
			Scenario 2			
Window	0-80	80-180	180-210	210-320	320-410	410-500
Length	80	100	30	110	90	90
β_P^S	0.39	0.16	0.34	0.17	0.26	0.39
β_P^A	0.43	0.18	0.27	0.09	0.23	0.43
β_P^A / β_P^S	1.11	1.09	0.80	0.53	0.89	1.11
v_1	0	0	0	0	150	300

The week evolution of the exposure (ς^S_{DOW} and ς^A_{DOW}) is assumed to be as follows: the exposure is at the reference level during the working days (Monday to Friday) for both symptomatic and asymptomatic people. It is assumed to be of very low level on Sunday because activities and thus interactions are low. They are assumed to be highly differentiated on Saturday with very low values for the symptomatic people (who are assumed to keep their activity to the minimum) and very high values for asymptomatic people (whose activity is necessary and presumably safe).

Finally the parameter *m* is the fatality rate and *v* the number of vaccination per day.

Table B: Weekly characteristics of the social forcing.

Day of Week	Monday	Tuesday	Wednesday	Thursday	Friday	Saturday	Sunday
				Scenario 1			
ς^S_{DOW}	1.	1.	1.	1.	1.	2.0	0.3
ς^A_{DOW}	1.	1.	1.	1.	1.	2.0	0.3
				Scenario 2			
ς^S_{DOW}	1.	1.	1.	1.	1.	2.0	0.3
ς^A_{DOW}	1.	1.	0.5	1.	1.	0.5	0.3

The SEi^2RVD model with weekly societal forcing (Eqs. 1) was run using the coefficients provided in Tables A-B in the present S2 Appendix and using the initial conditions $(S, E, i^S, i^A, R, V, D)_{t=0} = (5.10^7, 0, 0, 10, 0, 0, 0)$.

Input forcing and output time series are presented in Figs A and B for scenario 1 and Figs C and D for scenario 2 (both in the present S2 Appendix).

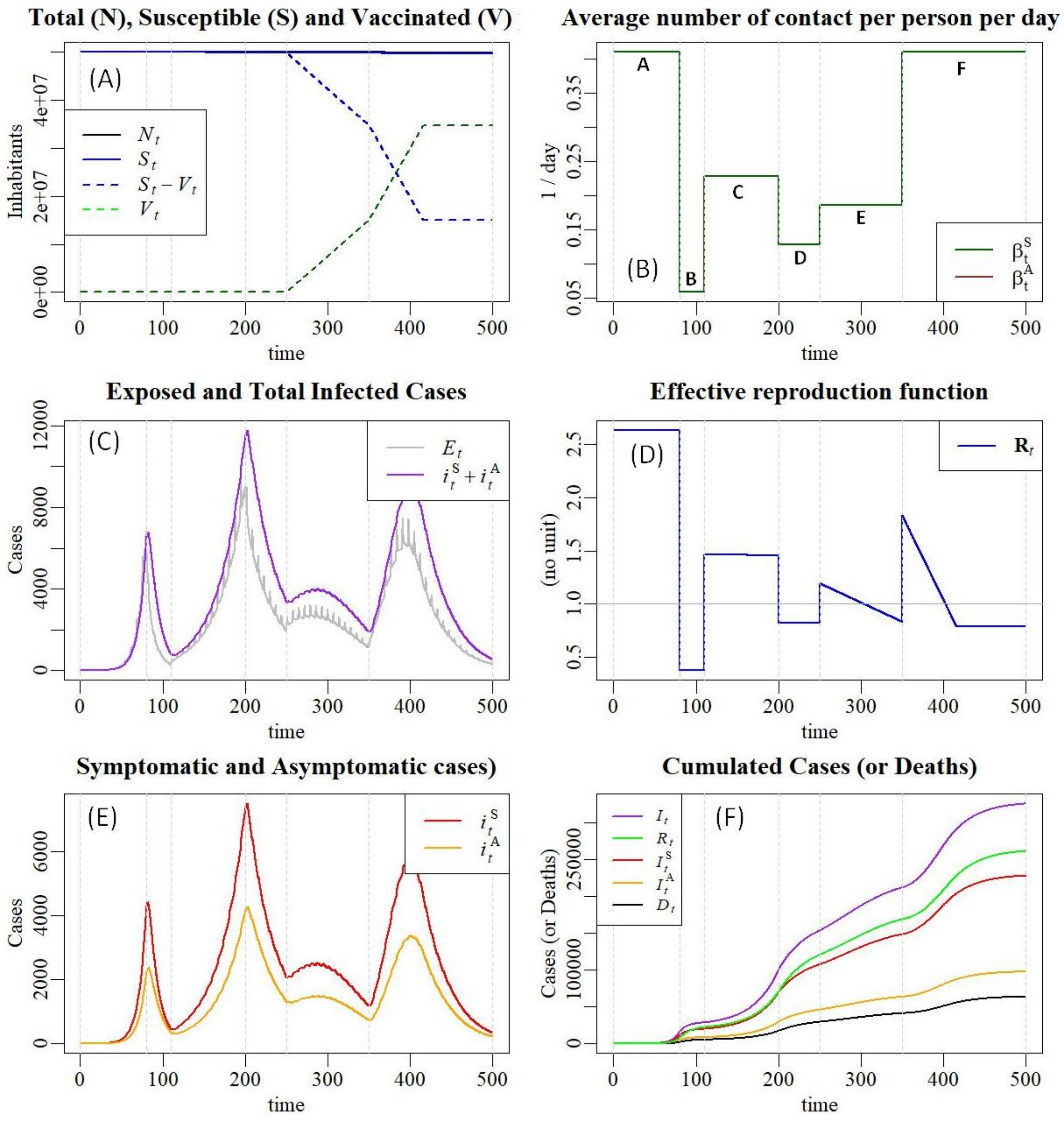

Figure A: Simulations run with the SEi^{2}RVD model (Eqs. 1 in the present S2 Appendix). Simulations of the total N_t, Susceptible S_t and Vaccinated V_t populations are presented in (A), the exposed E_t and the total infected populations i_t in (C), the symptomatic i_t^S and asymptomatic i_t^A infected population in (E) and the cumulated cases (total infected I_t, symptomatic I_t^S, asymptomatic I_t^A, and recovered R_t) and deaths D_t in (F). The variations of the average number of contact per person and per day is shown in (B) for both symptomatic β_t^S and asymptomatic β_t^A people, which are identical in the present scenario; the successive periods **A** to **F** of the scenario are explicitly mentioned and separated by vertical dashed lines (these vertical lines are also shown in the other plots). The effective reproduction number $\mathbf{R}_t$ is provided in (D).

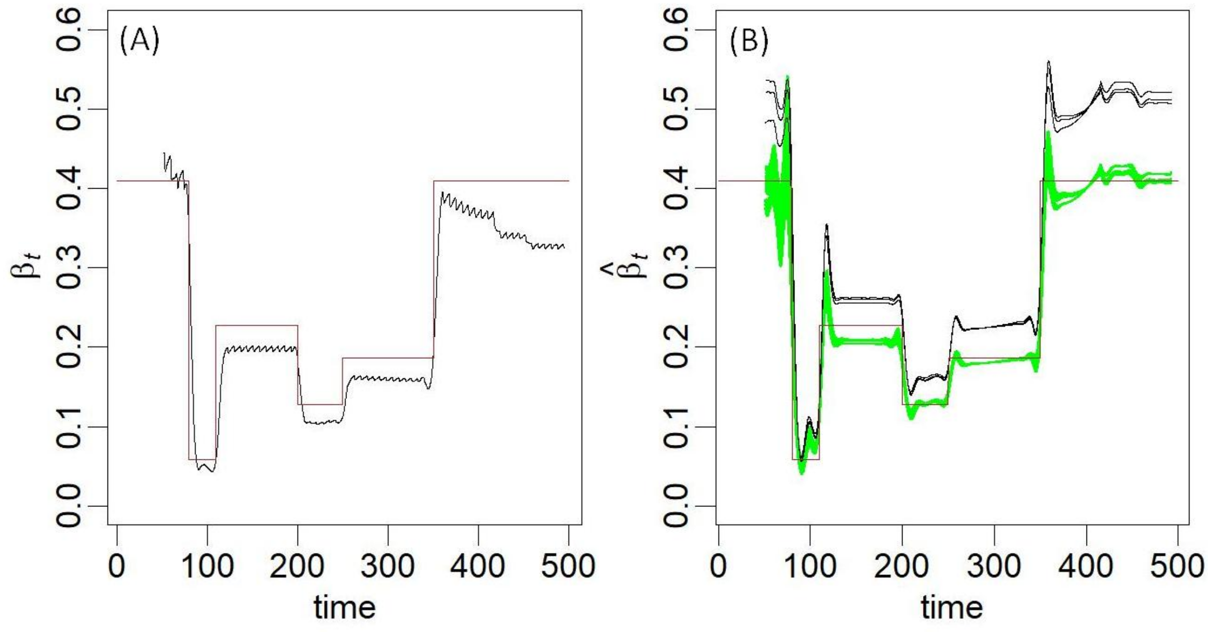

Figure B: Averaged contact number per person and per day estimated from synthetic time series I_t and i_t (Fig A) using Eq. 10 in S1 Appendix (in A) and from synthetic time series I_t only still using Eq. 10 in S1 Appendix, reconstructing i_t from I_t using Eq. 9 (in S1 Appendix), either with coefficient correction (Eq. 11 in S1 Appendix) $\xi = 0.75$ (in green) or not ($\xi = 1$) (in black) (in B). The original values β_t used in the scenario are also reported.

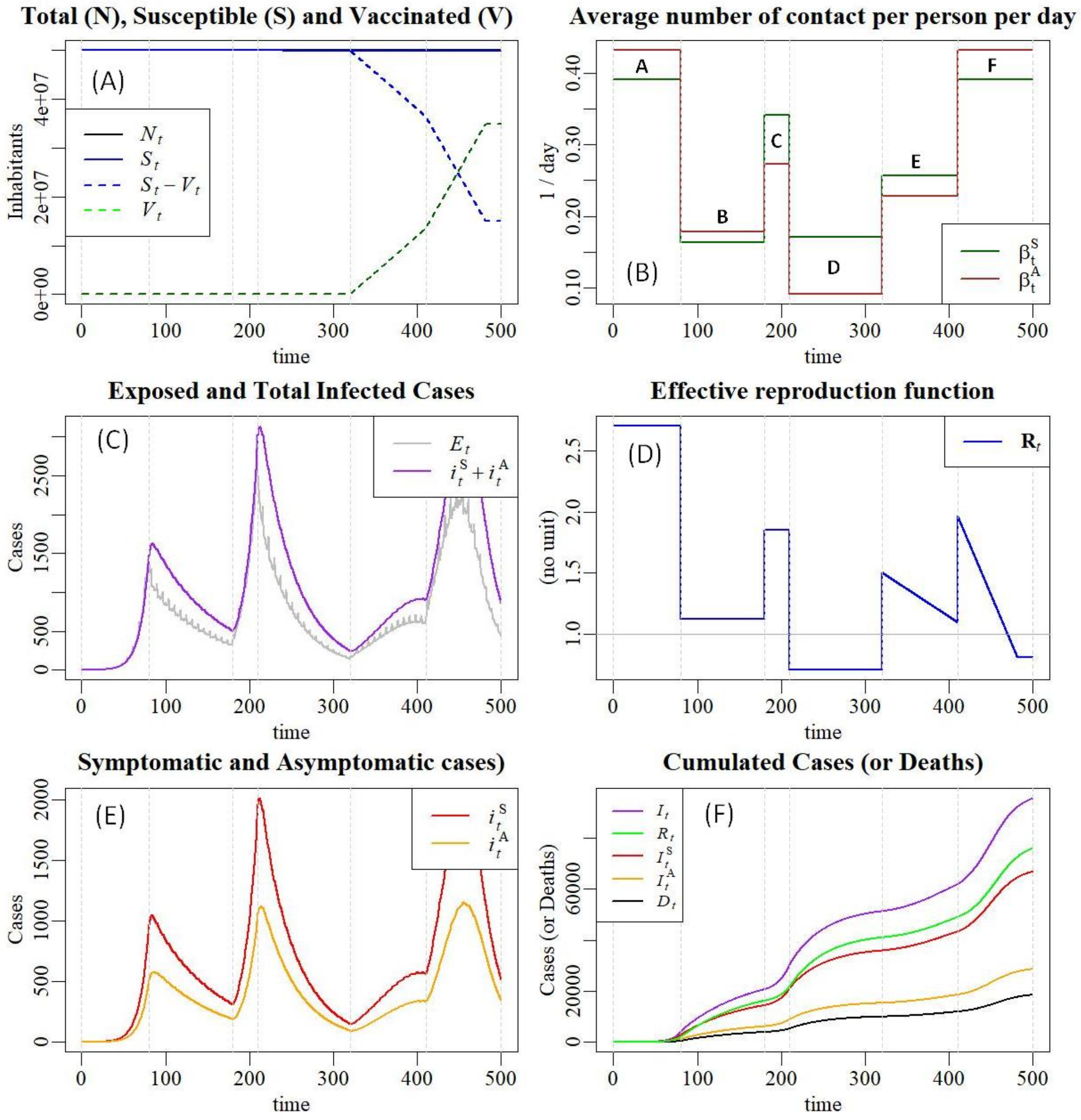

Figure C: Same as Fig A for scenario 2.

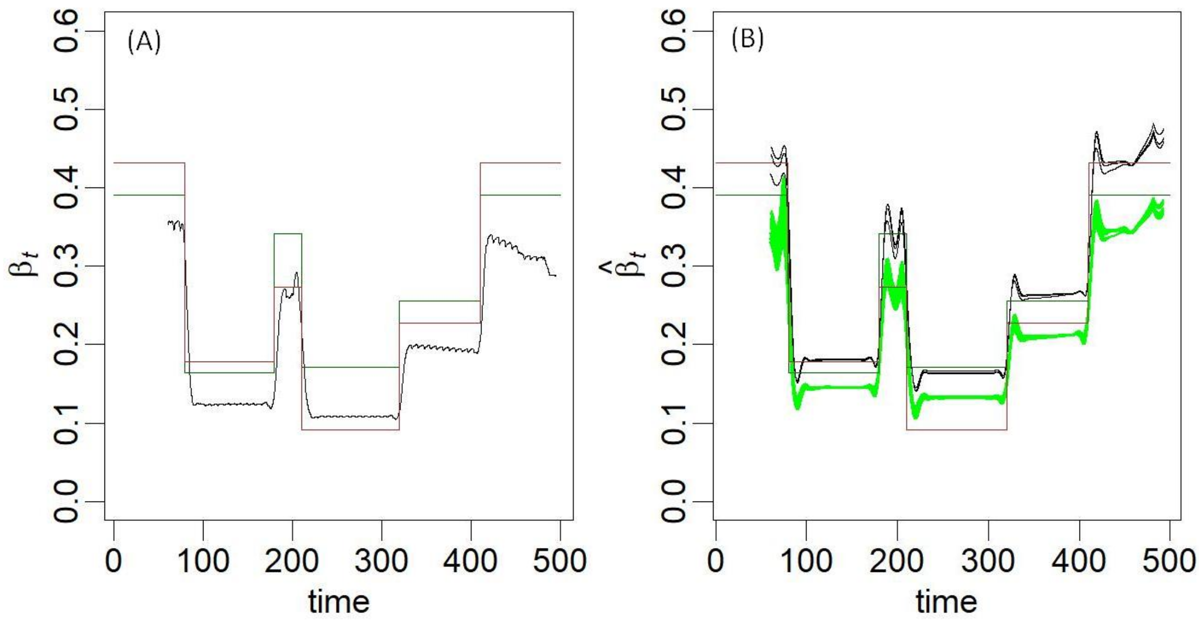

Figure D: Same as Fig C for scenario 2.

Reference

1. COVID-19 Pandemic Planning Scenarios, Centers for Disease Control and Prevention. (Consulted on 1 April 2021). [COVID-19 Pandemic Planning Scenarios | CDC]
