# Supplementary material for: COVID-19 in Africa: Underreporting, demographic effect, chaotic dynamics, and mitigation strategy impact
Source: PLoS Negl Trop Dis. 2022 Sep 16;16(9):e0010735. doi: 10.1371/journal.pntd.0010735 (PMC9518880; doi:10.1371/journal.pntd.0010735)
Supplement: S3 Appendix — All the global models obtained in the present study are based on the same general algebraic structure given in Eq 5 with n = 3. The detailed definition of the polynomial Q(X1, X2, X3) provided in the present Appendix are thus sufficient to have the full model description. Note that the initial conditions are also provided. (PDF) [file pntd.0010735.s015.pdf]

S3 Appendix for

Covid-19 in Africa: underreporting, demographic effect, chaotic dynamics, and mitigation strategies impact

Authors: Nathan Thenon[1,2†], Marisa Peyre[2], Mireille Huc[1], François Roger[2], Sylvain Mangiarotti[1*†].

[*]To whom correspondence should be sent: sylvain.mangiarotti@ird.fr

†These authors contributed equally to this work

Appendix S3 - Models equations

All the global models obtained in the present study are based on the same general algebraic structure given in Eqs. (5) (in the main manuscript), with $n = 3$. It is thus sufficient to provide the detailed definition of the polynomial $Q(X_1, X_2, X_3)$ to have the full model definition.

1. Cases models

The polynomials directly obtained for cases dynamics are given hereafter, with the initial conditions used in the simulations:

$$
\begin{aligned}
Q^I_{\text{Algeria}} = &+0.0796687009799944+0.0662341786995872\ I_3^2 - \kappa_1 \times 0.0796215959831427\ I_2 \\
&+0.038739626362673\ I_2 I_3+0.00436665937204388\ I_2^2 - 0.00226541691584844\ I_2^2 I_3 \\
&-0.000422658530759521\ I_2^3 - 0.00361610998969291\ I_1+0.00115155507457535\ I_1 I_3 \\
&-0.000428997637350344\ I_1 I_3^2+0.000928781337541139\ I_1 I_2 \\
&-0.000245339788878338\ I_1 I_2 I_3 - 2.04818368769602e\text{-}05\ I_1 I_2^2+3.93275783109323e\text{-}05 I_1^2 \\
&-1.04438432207189e\text{-}05\ I_1^2 I_3 - 2.24656357540239e\text{-}06\ I_1^2 I_2 \\
&-\kappa_2 \times 1.33664436257257e\text{-}07\ I_1^3
\end{aligned}
\tag{1}
$$

for $\kappa_1 = \kappa_2 = 1$, with $(I_1, I_2, I_3)_0 = (1.32140219, 0.40622939, 0.09728182)$.

$$
\begin{aligned}
Q^I_{\text{Côte d'Ivoire}} = &-0.109797306029186 I_3+0.0205209287472364 I_3^2 - 0.0868905890387966 I_2 \\
&+0.00227419260860002 I_2 I_3 - 0.00256777264961512 I_2^2 - 5.64800118265148e\text{-}05 I_2^3 \\
&+0.00437757534400393 I_1 I_3 - 0.000206522894087883 I_1 I_3^2 + 0.000657379569145025 I_1 I_2 \\
&-1.00282332259123e\text{-}05 I_1 I_2 I_3 + 2.41794779500537e\text{-}05 I_1 I_2^2 + 3.73062321820389e\text{-}05 I_1^2 \\
&-2.16893269336703e\text{-}05 I_1^2 I_3 - 3.28208395744655e\text{-}06 I_1^2 I_2 - 2.26305046278701e\text{-}07 I_1^3
\end{aligned}
\tag{2}
$$

with $(I_1, I_2, I_3)_0 = (3.78019267, 1.03816738, 0.09672889)$.

$$
\begin{aligned}
Q^I_{\text{Egypt}} = &+0.347551915847867 I_3^2 - 0.102945522563235 I_3^3 - 0.323684676282691 I_2 \\
&+0.236049232447228 I_2 I_3 - 0.0251438275023693 I_2 I_3^2+0.00623343603702519 I_2^2 \\
&-0.0065429655298832 I_2^2 I_3+\kappa \times 0.00537960774185894 I_1 - 0.0032743965335608 I_1 I_3 \\
&-0.00165176991509053 I_1 I_3^2 + 0.00661374370806083 I_1 I_2 - 0.00187710176207968 I_1 I_2 I_3 \\
&-7.27100800119011e\text{-}05 I_1^2+2.48290326239702e\text{-}05 I_1^2 I_3 - 3.76578599389823e\text{-}05 I_1^2 I_2 \\
&-2.04103975309291e\text{-}08 I_1^3
\end{aligned}
\tag{3}
$$

for $\kappa = 1$, and with $(I_1, I_2, I_3)_0 = (18.56026154, 0.54684471, -0.07842929)$.

$$Q^{I}_{\text{Ethiopia}} = +0.267967672783547 I_3^2 - 0.0390728732756903 I_3^3 - 0.0593653335905724 I_2$$
$$+0.0043915094574951 I_1 - \kappa \times 0.00106679691473617 I_1 I_3^2 + 1.18139802334684e\text{-}05 I_1 I_2^2 \quad (4)$$
$$-7.5725445491524e\text{-}05\ I_1^2 + 2.12654153118063e\text{-}07\ I_1^2$$

for $\kappa = 1$, and with $(I_1, I_2, I_3)_0 = (0.148557439, 0.007301641, –0.001853553)$.

$$Q^{I}_{\text{Ghana}} = -0.460640870877873\ I_3 - 0.0106389881683115\ I_3^2 - 0.164427462210515\ I_2$$
$$+\ 0.0398049970486589\ I_2 I_3 - 0.00203407583692159\ I_2 I_3^2 - 0.00010393348100922\ I_2^3$$
$$+\ 0.00413563939508601\ I_1 + 0.0055772972346767\ I_1 I_3 + 0.00162407387907056\ I_1 I_2$$
$$-0.000129625360168781\ I_1 I_2 I_3 + 6.48258295454445e\text{-}06\ I_1 I_2^2 \quad (5)$$
$$-3.55385837279337e\text{-}05\ I_1^2 - 1.2103505242464e\text{-}05\ I_1^2 I_3 - 3.38899598975704e\text{-}06\ I_1^2 I_2$$
$$+\kappa \times 3.71663071078492e\text{-}08\ I_1^3$$

with $\kappa = 1$ (applied to the last monomial) and with $(I_1, I_2, I_3)_0 = (1.5731661, 0.7370571, 0.2645352)$.

$$Q^{I}_{\text{Kenya}} = -0.518897751110126 - 0.00695195214544046 I_3^2 - 0.266696341079546 I_2$$
$$+0.0357899959683549\ I_2 I_3 - 0.00538122870953072 I_2 I_3^2 - \kappa_1 \times 40.0050226539649489 7 I_2^2$$
$$-0.000469295493196084\ I_2^3 + 0.0111278464207414 I_1 + 8.23468666450821e\text{-}06\ I_1 I_3^2 \quad (6)$$
$$+0.00283421917402513 I_1 I_2 - 0.000128470955256436 I_1 I_2 I_3 + 1.35062768144442e\text{-}05 I_1 I_2^2$$
$$-\kappa_2 \times 4.30872131966366e\text{-}05 I_1^2 - 4.80514923485185e\text{-}06 I_1^2 I_2 + 4.49423780653955e\text{-}08 I_1^3$$

for $\kappa_1 = \kappa_2 = 1$, with $(I_1, I_2, I_3)_0 = (63.007946, 1.062328, 1.041740)$.

$$Q^{I}_{\text{Libya}} = -0.0295999803766723 I_2 + 0.00451448056779914 I_1 - 5.37649858959794e\text{-}06 I_1^2 \quad (7)$$
$$-2.11264598883614e\text{-}08 I_1^2 I_2 + 1.5571629698307e\text{-}09 I_1^3$$

with $(I_1, I_2, I_3)_0 = (1.68428705, 0.17532753, -0.06714965)$.

$$Q^{I}_{\text{Namibia}} = +0.794849004986058 - 0.0137426747828601 I_3^2$$
$$-\kappa_1 \times 0.000880669916336909 I_3^3 - 0.138951149606636 I_2$$
$$-0.000315256792814862\ I_2 I_3^2 + 0.000919005556985513\ I_2^2 - 1.82934159221164e\text{-}05 I_2^3 \quad (8)$$
$$+0.000151489562878084 I_1 I_3 + \kappa_2 \times 0.000350599992671956 I_1 I_2$$
$$+1.97687227909123e\text{-}07 I_1 I_2^2 - 4.86511806983137e\text{-}06 I_2^2 - 1.03432015551895e\text{-}07 I_1^2 I_3$$
$$-1.59984646726366e\text{-}07 I_1^2 I_2 + \kappa_3 \times 2.38810765381376e\text{-}09 I_1^3$$

with $(I_1, I_2, I_3)_0 = (3.6059480, 0.6592280, 0.3532457)$.

$$
\begin{aligned}
Q^{I}_{\text{Nigeria}} &= +0.0343333345190545 - 0.0756396775696497 I_3^2 - 0.1103200282223132 I_2 \\
&+0.0339922989190682 I_2 I_3 + 0.0101452563456526 I_2^2 - 0.00366293002327367 I_2^2 I_3 \\
&-0.000934181818872205 I_2^3 - 0.00237756302125567 I_1 + 0.00115450490243978 I_1 I_3 \\
&+0.00097091291275902 I_1 I_3^2 + 0.00223618041065029 I_1 I_2 - 0.000241108286292507 I_1 I_2 I_3 \\
&-8.80125486240076e\text{-}05 I_1 I_2^2 + 3.48526219172797e\text{-}05 I_1^2 - 5.20379261720431e\text{-}06 I_1^2 I_3 \\
&-1.1683216302048e\text{-}05 I_1^2 I_2 - 1.34218095134093e\text{-}07 I_1^3
\end{aligned}
\tag{9}
$$

with $(I_1, I_2, I_3)_0 = (0.12468916, 0.04379826, 0.00517157)$.

$$
\begin{aligned}
Q^{I}_{\text{Senegal}} &= +0.0732315517322414 + 0.0540579926797526 I_3^2 - 0.00586775369583505 I_3^3 \\
&-0.0640643754193143 I_2 + 0.00526800398265657 I_2 I_3 - 0.00397448103442021 I_2 I_3^2 \\
&+0.00306541560044303 I_2^2 - 0.000464330034435878 I_2^3 - 0.00315639102570492 I_1 \\
&+0.000674573931781396 I_1 I_3 - 0.000226150093680181 I_1 I_3^2 + 0.000292153420066769 I_1 I_2 \\
&-5.78601736293428e\text{-}06 I_1 I_2^2 + 2.53714446410718e\text{-}05 I_1^2 - 2.48578957003552e\text{-}06 I_1^2 I_3 \\
&-4.92184930500184e\text{-}08 I_1^3
\end{aligned}
\tag{10}
$$

with $(I_1, I_2, I_3)_0 = (3.46637146, 0.64073597, 0.0833453)$.

$$
\begin{aligned}
Q^{I}_{\text{South Africa}} &= -0.126958415316111 I_2 - 1.55163089753807e\text{-}05 I_2^3 + 0.00329061175264698 I_1 \\
&+0.000298826837461897 I_1 I_2 + 3.93145619176576e\text{-}07 I_1 I_2^2 - 8.23576970097779e\text{-}06 I_1^2 \\
&-1.02521696340842e\text{-}07 I_1^2 I_2 + 2.81850646768005e\text{-}09 I_1^3
\end{aligned}
\tag{11}
$$

with $(I_1, I_2, I_3)_0 = (5.2297639, 2.4304931, 0.7082607)$.

$$
\begin{aligned}
Q^{I}_{\text{Zimbabwe}} &= -0.0900967854717015 I_3 - 0.00661602029774296 I_3^3 - 0.0491406408270159 I_2 \\
&-0.00255290659893318\ I_2 I_3^2 - 0.000541138403495552\ I_2^2 I_3 - 0.000282909762030189 I_2^3 \\
&+0.00272797031585041\ I_1 I_3 - 0.000256283420644421\ I_1 I_3^2 - 5.10666100414135e\text{-}05 I_1 I_2^2 \\
&+7.23106520159802e\text{-}05\ I_1^2 - 4.8621558720886e\text{-}07 I_1^2 I_2
\end{aligned}
\tag{12}
$$

with $(I_1, I_2, I_3)_0 = (4.773708481, 0.365475634, 0.005784271)$.

Other dynamical behaviours were found for cases dynamics. For Ghana, a bistable regime was discovered when tuning Eq. (5) with $\kappa = 0.85$, giving rise to two separated attractors (see Fig 2D in the main manucript) when starting from initial conditions $(I_1, I_2, I_3)_0 = (1.5731661, 0.7370571, 0.2645352)$ (in magenta), or $(I_1, I_2, I_3)_0 = (400, 0, 0)$ (in green).

Interestingly, the polynomial

$$
\begin{aligned}
Q^{I}_{\text{Ghana2}} &= -0.387949459699332 I_3 - 0.155078962631704 I_2 + 0.0337744581932721 I_2 I_3 \\
&- 0.00195768348928947 I_2 I_3^2 - 0.000121195029459481 I_2^3 + 0.00584450570536255 I_1 \\
&+ 0.00389337406184227 I_1 I_3 + 0.00153230608377978 I_1 I_2 - 0.000109216307590714 I_1 I_2 I_3 \\
&+ 6.59866420494425e\text{-}06 I_1 I_2^2 - 5.85677377292846e\text{-}05 I_1^2 - 7.69090331373171e\text{-}06 I_1^2 I_3 \\
&- 3.05365823727568e\text{-}06 I_1^2 I_2 + 8.85135966396584e\text{-}08 I_1^3
\end{aligned}
\tag{13}
$$

very similar to the other model obtained for Ghana (same terms as Eq. (5) except the term in I_3^2 missing), enabled to reproduce the whole types of trajectories in a single chaotic attractor (Fig 2E in the main manucript), also with $(I_1, I_2, I_3)_0$ = (1.5731661, 0.7370571, 0.2645352).

For Kenya, other interesting dynamics could be obtained by tuning the parameters of Eqs (6). The more complex situation was obtained with the original equations (Fig. 2C in green, in the main manucript) with large oscillations around a central point associated with smaller oscillations around small values (100 cases per day) and large values (450 cases per day and 520 cases per day). With the parameterization $\kappa_1 = 1.08$ and $\kappa_2 = 0.9901$, the smaller oscillations were removed, leading large oscillations around a genus-1 torus (Fig 2C in red, in the main manucript); with $\kappa_1 = 1.$ and $\kappa_2 = 0.99802$, keeping the large oscillations, the small oscillations around 450 cases per day were fostered (not shown); with $\kappa_1 = 1.$ and $\kappa_2 = 0.9882353$, only small oscillations around 500 cases per day were kept, slowly converging to a period-1 attractor around this value (Fig 2C in blue, in the main manucript). The same initial conditions with $(I_1, I_2, I_3)_0$ = (63.007946, 1.062328, 1.041740) were used in all the cases.

For Algeria, it was found possible to modify the average value of the periodic cycle at the convergence by tuning parameter κ_2 of Eq. (1) (see Fig 2B in the main manucript). A quicker propagation of the epidemic is obtained for $\kappa_2 = 1.2$ (oscillations around 250 cases per day, in blue), a slower (but with larger oscillations) one for $\kappa_2 = 0.75$ (average around 150 cases per day, in magenta). For $\kappa_1 = 1.7$, a very slow convergence to a period-1 cycle (in brown) was obtained through a nonchaotic toroidal transient (in cyan).

For Egypt, variations of parameter enabled κ in Eq. (3) enabled to develop a phase non coherent dynamics (with $\kappa = 1.7$, Fig 2A in green, in the main manucript), or to reduce it (with $\kappa = 1.25$, Fig 2A in magenta, in the main manucript).

2. Deaths models

The polynomials directly obtained for deaths are given hereafter:

$$
\begin{aligned}
Q^{D}_{\text{Algeria}} &= -\kappa \times 0.190661009721399 D_2 - 0.00288901208813148 D_1 \\
&+ 0.103827829105124 D_1 D_2 + 0.00199212706187995 D_1^2 \\
&- 0.0180987589643191 D_1^2 D_2 - 0.000314923292730593 D_1^3
\end{aligned}
\tag{14}
$$

for $\kappa = 1$, and with $(D_1, D_2, D_3)_0$ = (2.350603100, 0.090248589, –0.004403239).

$$
\begin{aligned}
Q^{D}_{\text{Cameroon}} &= +12.7808324888061 D_3^2 - 19.8284672476195 D_3^3 - 0.269460409652003 D_2^2 \\
&- 1.27015254177009 D_2^3 + 0.0825216825254995 D_1 D_3 - 2.88440011271812 D_1 D_3^2 \\
&+ 0.22971550408166 D_1 D_2 D_3 + 0.117082067115412 D_1 D_2^2 + 0.000734004211241529 D_1^2 \\
&- 0.0286453291499263 D_1^2 D_3 - 0.000273285434912587 D_1^3
\end{aligned}
\tag{15}
$$

with $(D_1, D_2, D_3)_0 = (1.01698111, 0.09243926, 0.01258062)$.

$$
\begin{aligned}
Q^{D}_{\text{Côte d'Ivoire}} &= -0.00159656293370687 + 0.152839563029428 D_3 - 9.09699927450249 D_3^2 \\
&- 0.208631827155974 D_2 - 2.50158590455907 D_2 D_3 - 278.888824651496 D_2 D_3^2 \\
&+ 5.50196226099524 D_2^2 - 167.400865912334 D_2^2 D_3 - 31.1644481409565 D_2^3 \\
&+ 0.0104571375363452 D_1 - 0.748276878762981 D_1 D_3 + 10.6568675409104 D_1 D_3^2 \\
&+ 0.641120512140422 D_1 D_2 - 0.998951052405393 D_1 D_2 D_3 - 4.9311392025704 D_1 D_2^2 \\
&- 0.0245513340513308 D_1^2 + 0.685581078491185 D_1^2 D_3 - 0.433808549960391 D_1^2 D_2 \\
&+ 0.014485432350422 D_1^3
\end{aligned}
\tag{16}
$$

with $(I_1, I_2, I_3)_0 = (0.79664811, 0.027177057, 0.001491929)$.

$$
\begin{aligned}
Q^{D}_{\text{Egypt}} &= +0.00360424207981085 - 0.196495303672811 D_3 + 3.16277068848196 D_3^2 \\
&- 1.1786088817269 D_3^3 - 0.193903628313008 D_2 + 1.54974618784215 D_2 D_3 \\
&- 4.14049520195359 D_2 D_3^2 - 0.131610160457468 D_2^2 + 0.525723366869267 D_2^2 D_3 \\
&- 0.126769464395039 D_2^3 - 0.00121898342983667 D_1 + 0.106333348895004 D_1 D_3 \\
&- 0.32858057170231 D_1 D_3^2 + 0.0609719574123028 D_1 D_2 - 0.260890028525343 D_1 D_2 D_3 \\
&+ 0.0264461215125738 D_1 D_2^2 + 0.000122930884429469 D_1^2 - 0.0116361672960978 D_1^2 D_3 \\
&- 0.00530611549106901 D_1^2 D_2 - 1.32225338943395e\text{-}05 D_1^3
\end{aligned}
\tag{17}
$$

with $(D_1, D_2, D_3)_0 = (0.058557124, 0.014613524, 0.003019658)$.

$$
\begin{aligned}
Q^{D}_{\text{Libya}} &= +0.534324550933693 D_3 - 0.0724075856532136 D_3^3 - 0.0787307026820384 D_2 \\
&+ 0.0444095960603132 D_2 D_3 + 0.0248069712372749 D_2^2 + 0.00520710316088048 D_1 \\
&- 0.0501062211581986 D_1 D_3 - 0.00244961892498698 D_1 D_2 \\
&- 0.0022093702169076 D_1 D_2 D_3 - 0.000969871105595799 D_1 D_2^2 \\
&- 0.000454394753471303 D_1^2 + 0.00114054290614677 D_1^2 D_3 + 1.06162147077378e\text{-}05 D_1^3
\end{aligned}
\tag{18}
$$

with $(D_1, D_2, D_3)_0 = (0.495186801, 0.030903699, 0.001010042)$.

$$
\begin{aligned}
Q^{D}_{\text{Namibia}} &= +0.5587307916100 19 D_3 - 1.53328090439678 D_3^3 + 0.0589878992077652 D_2^2 \\
&- 0.0320068007156988 D_2^3 - 0.119662495432852 D_1 D_3 - 0.00717005062822875 D_1 D_2^2 \\
&- 0.00036681528786263 D_1^2 + 0.00566621343689842 D_1^2 D_3 \\
&- 0.000157851160666792 D_1^2 D_2 + 3.54620325513566e\text{-}05 D_1^3
\end{aligned}
\tag{19}
$$

with $(D_1, D_2, D_3)_0$ = (0.59192824, 0.19602412, 0.02033359).

$$Q^D_{\text{South Africa}} = +0.00567748987859232 - 0.0407965458122204 D_2 - 0.00163361617685516 D_1 + 0.00102963245050581 D_1 D_2 + 4.79804286665403e\text{-}05 D_1^2 - 1.10988078532591e\text{-}05 D_1^2 D_2 - 3.16206776520503e\text{-}07 D_1^3 \quad (20)$$

with $(D_1, D_2, D_3)_0$ = (16,3012378, 1.0437449, 0.3989576).

$$Q^D_{\text{Tunisia}} = -0.0726344932529531 D_2 + 0.0013053835982996 D_1 + 0.00540995731970615 D_1 D_2 - 6.75920327930758e\text{-}05 D_1^2 - 0.000130898642568653 D_1^2 D_2 + 8.50605267050244e\text{-}07 D_1^3 \quad (21)$$

with $(D_1, D_2, D_3)_0$ = (0.98943082, 0.06020075, 0.01332138).

$$Q^D_{\text{Zimbabwe}} = +0.0926920347949401 D_3 + 0.835735156045351 D_3^2 - 0.718300162722018 D_3^3 - 0.143012408490209 D_2 + 0.187747965940508 D_2 D_3 - 0.33430941808173 D_2 D_3^2 + 0.0504419205144352 D_2^2 - 0.0480405031882636 D_2^2 D_3 - 0.0211663129285957 D_2^3 - 0.00199370398539795 D_1 - 0.0219149445790986 D_1 D_3 - 0.00883025440665343 D_1 D_3^2 + 0.0148961187703786 D_1 D_2 - 0.0019337819406716 D_1 D_2 D_3 - 0.00181912359250932 D_1 D_2^2 - 4.24281198206337e\text{-}05\ D_1^2 + 0.000910705108492471 D_1^2 D_3 - 0.000117812354585106 D_1^2 D_2 + 6.57330150088683e\text{-}06 D_1^3 \quad (22)$$

with $(D_1, D_2, D_3)_0$ = (0.306066088, 0.029837267, 0.00810896).

Other dynamical behaviours were found for deaths dynamics. For Algeria, the following model obtained:

$$Q^D_{\text{Algeria2}} = +0.0068330917922184 - 0.188512523788712 D_2 + 0.0795239378098587 D_2^2 - 0.0133918434091473 D_1 + 0.101782125167 62 D_1 D_2 - 0.0161682913315983 D_1 D_2^2 + 0.0064733944270254 D_1^2 - 0.0176403748649202 D_1^2 D_2 - 0.00087871337007575 D_1^3 \quad (23)$$

also with $(D_1, D_2, D_3)_0$ = (2.350603100, 0.090248589, –0.004403239). Interestingly, whereas the model based on Eq. (14) was developing slow and smoothed increases followed by decreases associated with large oscillations, this other one (Eq. 23) is developing slow and smooth decreases followed by large oscillations during the increasing period which suggest that these two behaviours are dynamically rather close one to another. Their dynamics is presented in Fig 2F in the main manucript.
