# Supplementary material for: COVID-19 in Africa: Underreporting, demographic effect, chaotic dynamics, and mitigation strategy impact
Source: PLoS Negl Trop Dis. 2022 Sep 16;16(9):e0010735. doi: 10.1371/journal.pntd.0010735 (PMC9518880; doi:10.1371/journal.pntd.0010735)
Supplement: S1 Table — The models dynamics was investigated considering 100 000 integration time steps of 0.1 day each (corresponding to a duration of 24 years). Metastable is mentioned when the integration could be checked on 20 000 time steps (∼6 years) only. P1, P2 and P5 refer to period cycles of period one, two and five, respectively. Toroidal chaos refers to chaotic attractors structured around, and bounded by, a toroidal structure (see [48] for details). (PDF) [file pntd.0010735.s016.pdf]

S1 Table for

# Covid-19 in Africa: underreporting, demographic effect, chaotic dynamics, and mitigation strategies impact

**Authors:** Nathan Thenon[1,2†], Marisa Peyre[2], Mireille Huc[1], François Roger[2], Sylvain Mangiarotti[1*†].

[*]To whom correspondence should be sent: sylvain.mangiarotti@ird.fr

†These authors contributed equally to this work

## Table S1. Dynamical regimes of the models.

**Table S1:** Dynamical regimes of the models. The dynamics was investigated considering 100 000 integration time steps of 0.1 day each (corresponding to a duration of ~24 years). Metastable is mentioned when the integration could be checked on 20 000 time steps (~6 years) only. P1, P2 and P5 refer to period cycles of period one, two and five, respectively. Toroidal chaos refers to chaotic attractors structured around a toroidal structure (see [1] for details).

| Country | Algeria | Cameroon | Côte d'Ivoire | Egypt | Ethiopia | Ghana | Kenya |
|---|---|---|---|---|---|---|---|
| *I* | P1 | Ø | P1 | Chaos | Chaos | Bistable chaos | Metastable chaos |
| *D* | Toroidal chaos | Chaos | P2 | Metastable chaos | Ø | Ø | Ø |
| **Country** | **Libya** | **Namibia** | **Nigeria** | **Senegal** | **South Africa** | **Tunisia** | **Zimbabwe** |
| *I* | Ø | Chaos | P1 | P1 | Metastable chaos | Ø | P5 |
| *D* | Torus | Chaos | Ø | Ø | Torus | Torus | Chaos |

## Reference.

1. Mangiarotti S, Letellier C, Topological characterization of toroidal chaos: A branched manifold for the Deng toroidal attractor, Chaos: An Interdisciplinary Journal of Nonlinear Science 2021; 31:013129. doi: 10.1063/5.0025924.
