# Supplementary material for: COVID-19 in Africa: Underreporting, demographic effect, chaotic dynamics, and mitigation strategy impact
Source: PLoS Negl Trop Dis. 2022 Sep 16;16(9):e0010735. doi: 10.1371/journal.pntd.0010735 (PMC9518880; doi:10.1371/journal.pntd.0010735)
Supplement: S2 Table — Models forecasting performances: forecasting error (in % of the maximum value observed during the modelling period), for a 10 day prediction horizon (90% confidence level) of daily cases and deaths per million inhabitants. Error level is provided for the validation window and also (in brackets) for the modelling window. The modelling and validation (in brackets) period length (in day) are also provided. (PDF) [file pntd.0010735.s017.pdf]

S2 Table for

Covid-19 in Africa: underreporting, demographic effect, chaotic dynamics, and mitigation strategies impact

Authors: Nathan Thenon[1,2†], Marisa Peyre[2], Mireille Huc[1], François Roger[2], Sylvain Mangiarotti[1*†].

[*]To whom correspondence should be sent: sylvain.mangiarotti@ird.fr

†These authors contributed equally to this work

Table S2. Models forecasting performances.

Table S2: Models forecasting performances: forecasting error (in % of the maximum value observed during the modelling period), for a 10 day prediction horizon (90% confidence level) of daily cases and deaths per million inhabitants. Error level is provided for the validation window and also (in brackets) for the modelling window. The modelling and validation (in brackets) period length (in day) are also provided.

Countries	Algeria		Cameroon	Côte d'Ivoire	Egypt	Ethiopia	Ghana	
	1	2					1	2
$\% e^I_{\tau=10}$	7(7)	–	–	58(23)	218(64)	34(10)	15(28)	16(29)
T	98(366)	–	–	107(352)	105(138)	151(308)	247(212)	247(212)
$\% e^D_{\tau=10}$	12(14)	14(15)	29(11)	51(11)	9(13)	–	–	–
T	105(266)	105(266)	247(192)	251(200)	103(340)	–	–	–
Countries	Kenya	Libya	Namibia	Nigeria	Senegal	South Africa	Tunisia	Zimbabwe
$\% e^I_{\tau=10}$	29(11)	26(13)	55(8)	6(12)	10(13)	29(6)	–	125(18)
T	143(131)	170(281)	230(146)	103(360)	105(240)	268(192)	–	234(167)
$\% e^D_{\tau=10}$	–	62(18)	90(21)	–	–	5(19)	20(17)	3(7)
T	–	139(250)	243(104)	–	–	103(98)	111(185)	105(262)
